# Intestinal microbiome interactions influence *Metarhizium*-based biocontrol efficacy against the sugar beet weevil

**DOI:** 10.1038/s41598-026-36038-8

**Published:** 2026-01-13

**Authors:** Daniela Wöber, Matthias Wernicke, Francisco Cerqueira, Katharina Wechselberger, Karin Hansel-Hohl, Stephan Manhalter, Eva M. Molin

**Affiliations:** 1 Center for Health & Bioresources, Unit Bioresources, AIT Austrian Institute of Technology GmbH, Tulln, Austria; 2 Institute of Computational Biology, University of Natural Resources and Life Sciences, Vienna, Austria; 3 Institute for Sustainable Plant Production (NPP), Austrian Agency for Health and Food Safety (AGES), Vienna, Austria

**Keywords:** Sex-based biological control, Entomopathogenic fungi, Microbe-microbe interactions, Intestinal microbiome, Microbiology, Zoology

## Abstract

The sugar beet weevil is considered one of the most economically important insect pests in sugar beet cultivation. A promising biological control strategy involves the natural interaction between entomopathogenic fungi and arthropods. The successful application of *M. brunneum* as part of integrated biological control strategies against the sugar beet weevil has already been demonstrated resulting in lethal mycosis. However, the efficacy of this strain is affected by multiple factors. The intestinal microbiome of insects harbours beneficial microbes that possess various functions, such as defence mechanisms against insect-pathogens. Thus, investigating intestinal microbial interactions in combination with *Metarhizium*-application could reveal microbes that modulate susceptibility to pathogens. This study investigated whether intestinal microbial interactions influence mycosis caused by *M. brunneum* and *M. robertsii*. We analysed the intestinal microbiome of both treated and untreated sugar beet weevils, distinguishing between mycotic and non-mycotic individuals at the time of death. Notably, *Pantoea* and *Enterobacter* were significantly associated with mycotic individuals and may act as a potential antagonist to *Metarhizium*. In contrast, healthy individuals harboured diverse microbial communities that may provide a protective barrier against entomopathogens. However, the intestinal microbiome of non-mycotic specimens also comprised genera with presumed insecticidal properties, including *Serratia*, *Penicillium* and *Cladosporium*. The last two were also observed in the intestines of male individuals, which were generally at a higher risk of mortality. Further investigation is needed to confirm their insecticidal potential in the sugar beet weevil. A combined application could improve the efficacy of *Metarhizium*-based biocontrol, contributing to more sustainable pest management strategies.

**Supplementary Information:**

The online version contains supplementary material available at 10.1038/s41598-026-36038-8.

## Introduction

Sugar beet (*Beta vulgaris* ssp. *vulgaris* var. *altissima* Döll) is a crop of high economic value, serving as a major source for sugar production, and an important export in many European countries^1^. Maintaining yield quality and quantity is of great importance to producers. Despite the implementation of plant protection measures, around a quarter of yield losses are attributed to plant diseases and pests each year^2^. Among the most relevant insect pests is the sugar beet weevil (SBW) (*Asproparthenis punctiventri*s Germar, formerly *Bothynoderes punctiventris* Germar, Coleoptera: Curculionidae), a widespread species in Eurasian countries, including Austria^3^. Naturally, young SBWs emerge from April to June and migrate to adjacent sugar beet fields, where they feed on young leaves^4,5^. It is well known, that one single SBW can destroy up to 50% of sugar beet plants at the cotyledon stage per square meter^6^. Global warming and increasing temperatures are additionally accelerating insect morphogenesis and feeding activity^7^. In the worst-case scenario, this results in pest calamities that significantly reduce farmers’ yield potential. In previous years, pest management involved the use of neonicotinoids to control damage at low weevil population levels. However, due to their broad-spectrum activity against Homoptera, Coleoptera and Lepidoptera^8^, neonicotinoids were banned in the EU in 2018, leaving sugar beet cultivation without any sustainable pest management solutions^6,9^. Ultimately, effective pest control methods against the SBW remain to be investigated and successfully implemented.

Considering that fungi are the most common natural pathogens of arthropods, accounting for around 60% of diseases, entomopathogenic fungi have great potential as biological control agents against insect pests^10,11^. Insect mortality is caused by a combination of nutrient deficiency, fungal toxicity, circulatory system disorders and internal organ damage^11^. Entomopathogenic fungi exhibit a range of ecological functions beyond insect-pathogenicity, as they can also be plant endophytes, antagonists of plant diseases, plant growth promoters and rhizosphere colonizers^10^. *Metarhizium* Sorokin (Hypocreales: Clavicipitaceae) is a well-known genus of entomopathogenic fungi, which has already demonstrated its potential of controlling various pests such as the rice planthopper (*Sogatella furcifera*^12^), the northern root-knot nematode (*Meloidogyne hapla*^13^), and the migratory locust (*Locusta migratoria*^14^). Recently, *M. brunneum* has shown promising results in controlling adult SBWs^15^. Similarly, *M. robertsii* has been described as suppressing the larval development of the black cutworm (*Agrotis ipsilon*^16^).

The virulence of entomopathogenic fungi depends on various factors including temperature, humidity, pH-level, spore quality and quantity, strain and host suitability^17^. In recent years, the microbiome of target organisms has come into focus as another potential factor influencing the infection process of entomopathogenic fungi^18,19^. In natural infections, multiple species of (entomopathogenic) fungi, bacteria, viruses, and nematodes coexist and interact through competition, suppression, and synergism^11^. Next to molecular defence mechanisms, the insect host’s microbiome and intestinal microbiota-host interactions are essential for its defence mechanisms and immune regulation^20,21^. Fungal growth can be prevented at the stage of penetration and host colonization if the fungus is unable to compete with the resident intestinal or cuticular microbiome that exhibit antifungal activity^19,22^. Defence mechanisms in the host’s intestinal microbiome against *M. brunneum* have already been observed in wireworms (*Agriotes* spp.^23^). Moreover, a significant reduction in the number of intestinal bacteria in the Colorado potato beetle (*Leptinotarsa decemlineata*) has also been reported to correlate with *M. robertsii*-induced mycosis^22^. However, microbially diverse environments also facilitate interactions between microbes that complement or enhance the function or activities of taxa^24,25^. Therefore, we hypothesize that both antagonistic and agonistic microbial taxa associated with *Metarhizium* spp. can be identified that correlate with successful mycosis in SBWs. These taxa could maximize the potential of entomopathogens as an alternative to conventional insecticides in sugar beet cultivation.

As current biological control strategies and related research predominantly focus on individual microbial species rather than on microbial networks, potential microbial interactions in active infestations remain to be understood. Additionally, the intestinal microbiome can strengthen the immune system and activate defence mechanisms against potential pathogens. Thus, our work builds on previous research investigating the application of *Metarhizium* as a biological control agent^13,22,23^, especially in the context of sugar beet protection^15^. Given that the insect intestinal microbiome can influence specific pest-plant interactions^19^, the focus in this study was broadened to include the SBW’s intestinal microbiome. Therefore, SBWs were treated with either *M. brunneum*, *M. robertsii*, or left untreated as a control group, until death. Amplicon sequencing of microbial gene regions was performed to identify intestinal microbial patterns associated with the presence or absence of *Metarhizium*-mycosis.

## Materials and methods

### Sampling sites

In 2023, adult SBWs (*n* = 80–100 per field) were collected from wheat fields where sugar beet had been cultivated the previous year, and the pest was known to be present. The sampling sites were Ruppersthal (48.456750, 15.934230), Obermallebarn (48.456920, 16.127930) and Rückersdorf (48.408610, 16.386860) in Lower Austria. As the emergence of beetles from overwintering in the soil depends on temperature and the depth at which they overwinter^26^, not all beetles come to the surface at the same time. From April to the end of May, when the first weevils emerged after hibernation and became active, beetles were regularly collected. Newly emerged SBWs were hand-collected and stored in opaque tubes at 5 °C (the developmental threshold for SBWs^27^), until a sufficient number of beetles per site was available to conduct the experiment.

### *Metarhizium*-treatment

SBWs were kept individually in ventilated plastic cages (9 × 6.5 × 5 cm; Neupack Verpackungen GmbH, Germany) within a climate chamber (25 °C, 60% RH, 14:10 h light-dark). They were fed fresh sugar beet (variety *Caroll*, Strube, Germany) three times per week (five to eight seedlings per SBW) and provided with water via a 5 mL Eppendorf tube containing a paper towel dispenser, refilled weekly.

After a seven-day quarantine period, 20 SBWs were selected from each of the three sampling sites and assigned to three treatments for the bioassay (*n* = 60 SBWs per treatment): *M. brunneum* (Ma 43 – BIPESCO 5/F 52), *M. robertsii* (GT 4), and a control group that received no fungal treatment. *M. brunneum* Ma 43 is a naturally occurring strain, which has been isolated from a codling moth in Austria^28^. *M. robertsii* (GT 4) was isolated and genotyped from soil in Styria, Austria^29^ and was found in mycotic SBWs collected in Lower Austria^15^.

## Bioassay and microbiome analysis

### Spore inoculation

Spore viability of *M. brunneum* and *M. robertsii* was assessed by applying 3 × 10 µL spore suspension to Sabouraud-2-glucose agar plates (10 g peptone, 20 g glucose, and 18 g agar). The plates were then incubated in the dark at 25 °C for 24 h. Germination (germ tubes exceeding the length of spores) was evaluated microscopically (400x, 100 spores per count). Average germination rates of both species exceeded 90%.

The stock culture was grown on Sabouraud-2-glucose at 25 °C for 10 days to obtain conidia. To prepare the spore suspension, the conidia were suspended in 10 µL of sterile water containing 0.1% (w/v) Tween 20 and scraped with a spatula. Conidial suspensions were then filtered through a funnel containing filter fleece to remove the mycelium. The conidial suspensions were counted using a haemocytometer and diluted to the desired concentration in sterile water containing 0.1% Tween 20.

The inoculation method was based on Zottele *et al*. (2023)^15^. In brief, SBWs were inoculated by pipetting 50 µL of the spore suspension (5 × 10^6^ spores per weevil and 0.1% Tween 20) directly onto the dorsal side of the body, between the pronotum and elytra to ensure contact between host and pathogen. The control group received 50 µL sterile water with 0.1% Tween 20. SBW vitality was checked daily. Death was defined as lack of response to mechanical stimulation. Non-responsive specimens after one hour were declared dead and stored in Eppendorf tubes at -35 °C until further examination.

### Intestinal tract extraction

**Fig. 1:**
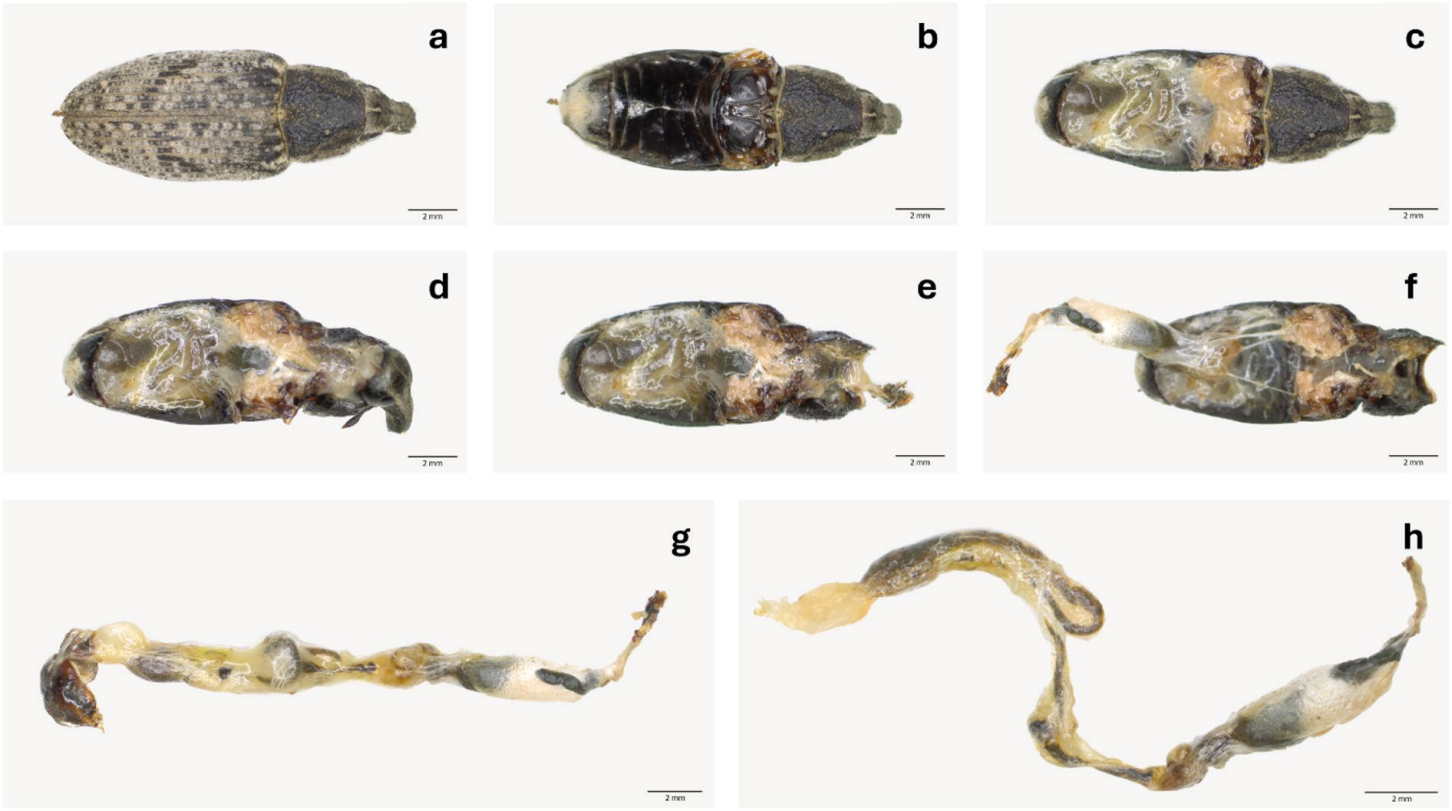
Extraction of intestinal tract for sampling of *A. punctiventris*. Removal of legs (**a**), elytra, wings (**b**), tergites (**c**), pronotum (**d**), head and rostrum (**e**) using forceps and micro scissors. Extraction of the intestinal tract by carefully pulling it out while holding the mouthparts (mandibles, maxilla, premetum, postmetum) with forceps (**f**). Separation of the intestinal tract (foregut, midgut and hindgut) from sclerotized parts (**g**; image is composed of two images). The anal plate, organs, and fat body were extracted using forceps. The mouthparts were detached by cutting the area anterior to the pharyngeal plate with micro scissors. The final intestinal tract is shown without the mouthparts and anal plate (**h**). Scale bar: 2 mm.

SBWs were surface-sterilized in 70% ethanol for 60 s and rinsed three times with sterile water. Instruments and dishes were sterilized with 96% ethanol. The intestinal tracts were dissected under a stereomicroscope (Olympus SZX10) using the procedure described in Fig. 1. Extracted intestinal tracts were transferred individually to 1.5 mL polypropylene microtubes (Semadeni, Germany) and stored at -35 °C for microbiome analysis. During dissection, the sex of each SBW individual was determined.

**Fig. 2:**
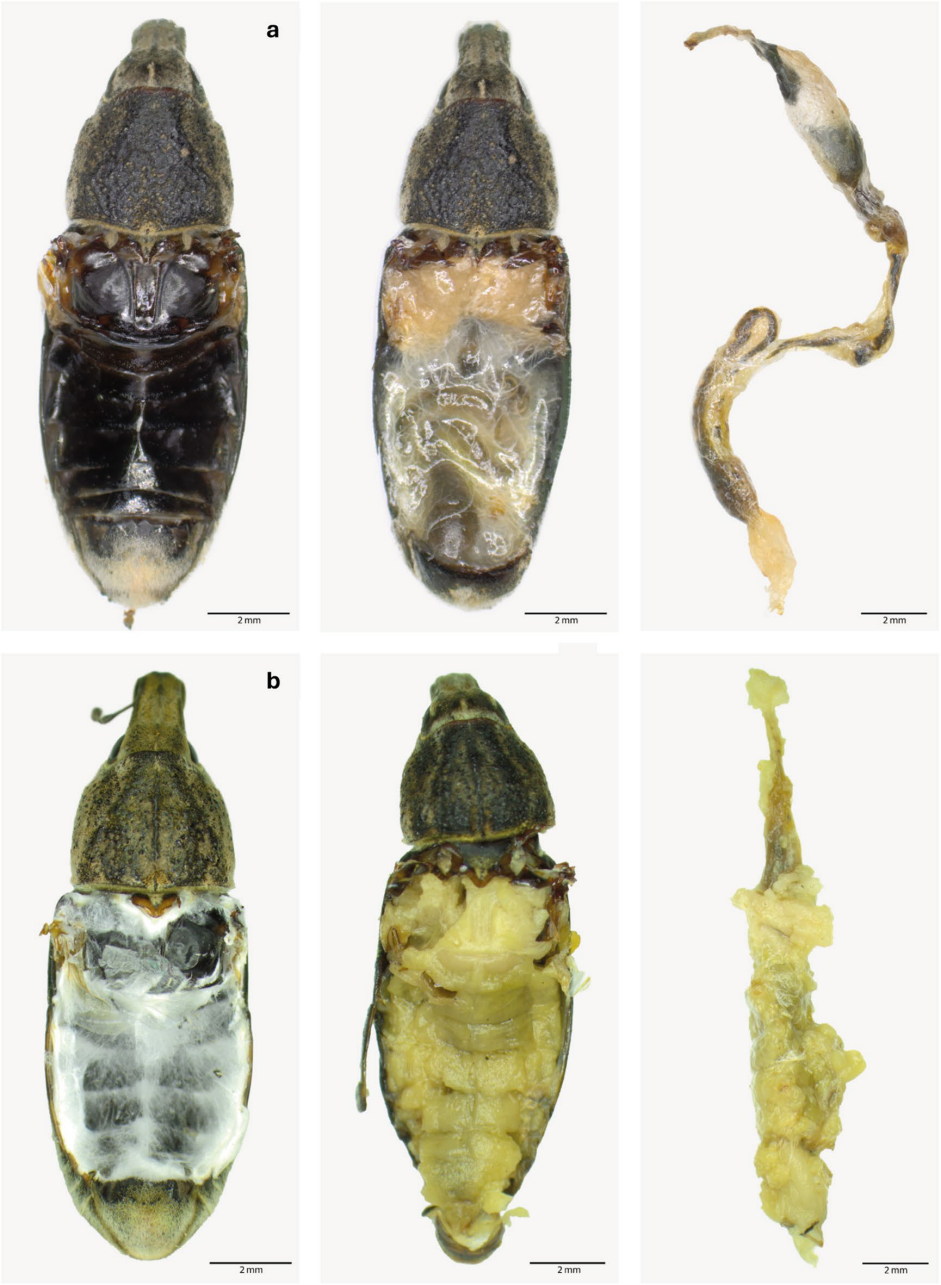
Comparison of body parts of non-mycotic and mycotic SBWs. The tergites are shown on the left, the inner organs in the centre, and the intestinal tracts with surrounding tissue on the right for healthy (non-mycotic) specimens (**a**) and mycotic individuals (**b**). Scale bar: 2 mm.

To confirm mycosis as the cause of death, dead SBWs were placed on water agar (2%) and incubated in the dark at 25 °C. After 14 days, mycosis was confirmed if *Metarhizium* spp. spore packets were visible under a stereomicroscope (Fig. 2). A distinction was made between external mycosis (externally visual mycosis) and internal mycosis (mycotic intestine).

### DNA extraction and amplicon sequencing

Intestinal tracts from 70 SBWs (*n* = 23–24 per treatment) were analysed individually per SBW sample, including all specimens with confirmed *Metarhizium-* mycosis (*n* = 22), the control group (*n* = 23), and treated but non-mycotic individuals. Intestines were lyophilized and ground in a Retsch mill (four minutes, 24 Hz, two steel balls). The DNeasy PowerSoil Pro Kit (Qiagen) was used following the instructions. DNA quality was assessed using Picogreen measurements.

For 16S amplicon sequencing, PCR was performed with primers Illumina_16S_341F (5´-TCGTCGGCAGCGTCAGATGTGTATAAGAGACAGCCTACGGGNGGCWGCAG-3´) and Illumina_16S_805R (5´-GTCTCGTGGGCTCGGAGATGTGTATAAGAGACAGGACTACHVGGGTATCTAATCC-3´). Each 25 µL reaction contained 5 µL 5x KAPA HiFi buffer with Mg, 0.5 µL 10 mM dNTPs (KAPA), 0.75 µL of each primer (10 µM), 0.25 µL KAPA HiFi polymerase, and 2 µL of template. Cycling conditions were: 95 °C for three minutes, 25 cycles at 98 °C for 20 min, 60 °C for 30 min, 72 °C for 30 min, and 72 °C for five minutes (Kapa HiFi Hot Start PCR Kit; Roche Diagnostics Deutschland GmbH). For ITS sequencing, forward “ITS3MIX” and reverse primer “ITS4MIX” were used. Detailed information of the primers and their sequences can be found in Wöber *et al*. (2025)^30^. The reaction contained 0.3 µL of each primer (10 µM) and 4 µL template. The cycling conditions were identical to 16S but run for 28 cycles. PCR products were verified on agarose gels.

A second PCR was used to index the libraries with the Nextera XT Index Kit. Each 50 µL reaction contained 10 µL of 5x KAPA HiFi buffer with Mg, 1.5 µL of 10 mM dNTPs (KAPA), 1.5 µL of each primer (10 µM), 0.25 µL of KAPA HiFi polymerase and 1 µL amplification product. Cycling conditions were: 95 °C for three minutes, 12 cycles at 95 °C for 30 min, 60 °C for 30 min, 72 °C for 30 min, and 72 °C for five minutes (Kapa HiFi Hot Start PCR Kit). Indexed products were visualized on 1.5% agarose gel, quantified with Image Lab v6.0.1 build 34 (Bio-Rad), and pooled. Libraries were purified with phenol/chloroform extraction, Amicon Ultra – 0.5 30 K Membran filters, and AMPure XP Reagent (Beckman Coulter). Library quantification was done using Nanodrop and Picogreen, and samples were normalized to 4 nM. Libraries were denatured with NaOH, diluted with hybridization buffer to 6 pM and heat denatured. Paired-end sequencing (2 × 300 bp) was carried out on an Illumina MiSeq using the v3 reagent kit, including 8% PhiX as internal control.

### Sequence processing

Illumina reads were filtered with Bowtie2 v2.4.2^31^ to remove PhiX contamination, and quality was checked with FastQC v0.11.9^32^. Primers were trimmed using Cutadapt v3.4^33^. Sequences were quality-filtered, trimmed and denoised; amplicon sequence variants (ASVs) were merged, and chimeras were removed using DADA2 v1.18.0^34^. Filtered fungal ASVs were screened using ITSxpress v1.1.3^35^. Taxonomic assignment of 16S rRNA gene and ITS ASVs was performed using the RDP classifier in DADA2 against SILVA v138.1^36^ and UNITE 8.3^37^, respectively.

ASV tables were imported into R-4.4.1^38^. Chloroplast and mitochondrial sequences were removed from 16S data. Samples with fewer than 1,000 reads were excluded (ASP_15 for both datasets and ASP_63 for the 16S dataset only), leaving 68 16S samples and 69 ITS samples. Taxa were filtered using OTUtable^39^, with persistence set to 1 and abundance to 0.01, reducing 16S taxa from 158 to 156; no ITS taxon was removed. Both datasets were subject to multiple rarefactions, repeated 1,000 times, with the minimum sample size as cutoff (rtk^40^). Resulting ASV tables were converted into phyloseq objects. An additional phyloseq object containing fungal taxa was created, excluding all *Metarhizium* species (161 ASVs instead of 187).

### Statistical and microbial diversity analysis

The bioassay was analysed using SPSS 26.0.0.1^41^. The differences in lifespan between the treatments, and between the mycotic and non-mycotic SBWs, were analysed using Kruskal-Wallis (KW) tests. A Kaplan-Meier (KM) analysis was performed using the R function survfit()^42^ to estimate differences in the survival probability per treatment group. The survival curves were visualised using ggsurvplot()^43^.

For intestinal bacterial 16S and fungal ITS data were analysed and interpreted in relation to mycosis affecting the entire body, excluding the intestines. Alpha diversity (Shannon index) of rarefied ITS and 16S data was calculated and visualized with ggplot2^44^. Differences in alpha diversity relating to treatments and presence of mycosis were calculated using Wilcoxon tests with pairwise.wilcox.test(). Beta diversity was calculated on centered log-ratio (CLR) transformed ASV tables (microbiome^45^). Community composition was analysed with permutational multivariate ANOVA (PERMANOVA) on an unweighted UniFrac distance matrix (vegan^46^), starting from a full model (incl. treatment, survival span, external and internal mycosis, sex and sampling location), followed by backward elimination of terms with the highest non-significant p-values. Ordination was performed using robust principal component analysis (RPCA^47^), and only variables retained as significant in the final PERMANOVA model were visualised in the RPCA plots.

Species composition was analysed using phyloseq^48^. Total abundances of fungal and bacterial families were plotted by treatment and survival span using ggplot2. ASVs at family-level were filtered with a minimum prevalence of 10% and a minimum total abundance of 1,000. The ten most abundant genera were extracted separately for mycotic and non-mycotic weevils (excluding the control group). ASVs at genus-level were transformed into relative abundances using total sum scaling calculated via transform_sample_counts(function(x){x/sum(x)}) and plotted via ggplot2.

### Microbiome association analysis

Differential abundance was done with DESeq2^49^ to identify distinct microbes at genus-level between mycotic and non-mycotic SBWs (without control group; non-mycotic SBWs as reference). Low-abundance taxa were filtered using prevalence thresholds of 10%. Taxa with log2 fold-change (log2FC) coefficients > |2| and p-values < 0.05 were considered significant. Fold changes of significant taxa were plotted using ggplot2, and relative abundances of indicator taxa were visualized with ComplexHeatmap^50^.

MaAsLin2^51^ was used to assess the associations between microbes and mycosis caused by either *M. brunneum* or *M. robertsii* (mycotic SBWs as reference), with the survival span set as random factor. The analysis was performed at the final taxonomic level of each ASV, with taxa filtered at 10% prevalence. Significant associations were selected based on coefficients > |2| and p-values < 0.05.

Phylogenetic heat trees were constructed using metacoder^52^. Data were subset based on fungal treatments (excluding the control group) to compare SBWs exposed to *M. brunneum* and *M. robertsii*. Analysis was done to assess microbial differences between mycotic and non-mycotic SBWs as well as between female and male individuals. Data were normalized using calc_obs_probs() and Wilcoxon Rank Sum tests were performed with compare_groups(). The median ratio of log2(x) values were visualized in red (mycotic) and blue (non-mycotic) and significance was determined.

## Results

### Successful *Metarhizium*-mycosis impacts SBW survival

**Fig. 3:**
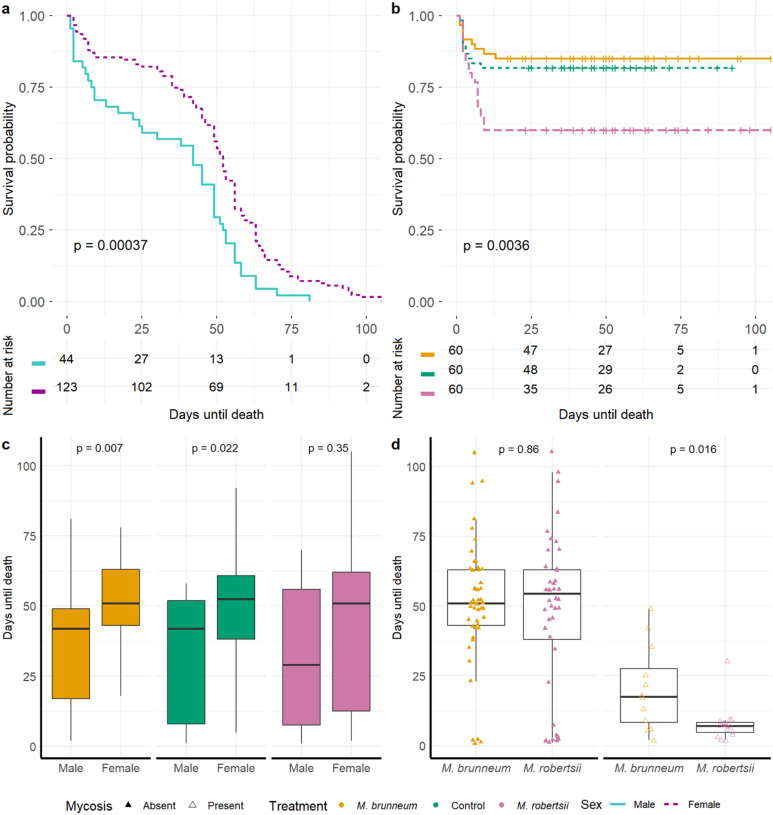
Bioassay results. Survival probability [%] based on KM tests is shown in line graphs comparing sex (turquoise: male; purple: female) (**a**) and treatment groups (yellow: *M. brunneum*; pink: *M. robertsii*; green: control group) (**b**). Mean lifespan (in days) of each SBW based on the treatment group and sex (**c**), and the presence or absence of a mycosis (filled triangle: non-mycotic; empty triangle: mycotic) caused by either *M. brunneum* or *M. robertsii* were determined (**d**). Significant differences in mean survival were determined using KW tests. NS = not significant, * *p* ≤ 0.1, ** *p* ≤ 0.05, *** *p* ≤ 0.01.

Approximately 20% of the weevils treated with *M. brunneum* and 27% treated with *M. robertsii* showed mycosis after death. In contrast, none of the deceased weevils in the control group exhibited *Metarhizium*-mycosis, thus the infection rates were significantly higher in fungal treatments (*p* < 0.001, KW). The sampling sites (Obermallebarn, Rückersdorf and Ruppersthal) had no effect on either the survival span or the number of mycotic individuals at the collection sites (*p* > 0.05, KW). Sexual dimorphism in terms of survival time was observed in SBWs, with a significantly shorter survival of male individuals (Fig. 3a and Supplementary File S1, Fig. S1a; *p* < 0.01). Significant differences in survival rates were observed between male and female SBWs treated with *M. brunneum* (*p* < 0.05, KW) or left untreated (control group of the bioassay only; Supplementary File S1, Fig. S1c; *p* < 0.05, KW), as well as a tendency in SBWs treated with *M. robertsii* (Fig. 3c). In general, treatment had an impact on the SBW longevity, with weevils infected by *M. robertsii* living significantly shorter than those infected by *M. brunneum* (*p* < 0.05, KM; Fig. 3b and Supplementary File S1, Fig. S1b). Median survival time for *M. brunneum* was 49 ± 1.927 days standard error (SE) (95% confidence interval [45.224; 52.776]), for *M. robertsii* 45 ± 7.746 SE (95% CI [29.818; 60.182]), and for the control 49 ± 2.903 SE (95% CI [43.310; 54.690]).

Almost all weevils infected with *M. robertsii* and exhibiting mycosis died within ten days after the fungus was applied (*n* = 15) (one individual died after 30 days). In contrast, one third of all SBWs that became mycotic following infection with *M. brunneum* did not survive the first ten days of the experiment (*n* = 3) (Fig. 3d, Supplementary File S1, Fig. S1d). The survival probability was significantly different for each treatment group in both the bioassay and the microbiome analysis (*p* < 0.01, KM) (Fig. 3b, Supplementary File S1, Fig. S1b). In both analyses, SBWs treated with *M. robertsii* were found to be at the greatest risk of mortality, with the fewest individuals surviving for 25 days.

For the following microbiome analysis, SBWs were categorized into three distinct groups according to their survival time: SBWs that died within the first ten days of the experiment were grouped into the “short” survival category (control: *n* = 1; *M. brunneum*: *n* = 3; *M. robertsii*: *n* = 11); those that survived for up to 50 days were categorized into “medium” (control: *n* = 13; *M. brunneum*: *n* = 13; *M. robertsii*: *n* = 5); and SBWs with a survival span of over 50 days were grouped into the “long” survival category (control: *n* = 9; *M. brunneum*: *n* = 8; *M. robertsii*: *n* = 7) (see Supplementary File S1, Fig. S1).

### Survival span of SBWs correlates with intestinal microbiome diversity

**Fig. 4:**
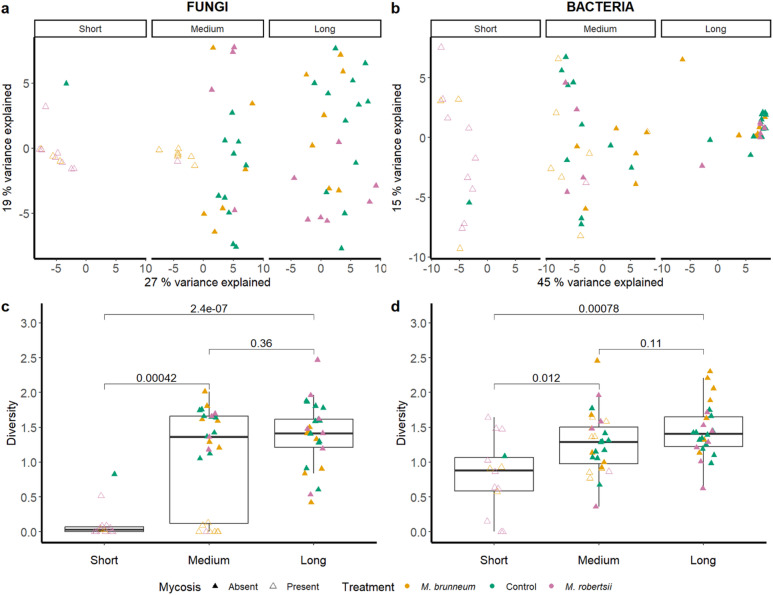
Fungal (**a**,** c**) and bacterial (**b**,** d**) beta and alpha diversity of the intestinal microbiome of SBWs. Diversity was analysed based on survival spans of SBWs (short: ≤ ten days; medium: ≤ 50 days; long: > 50 days), treatments (yellow: *M. brunneum*; pink: *M. robertsii*; green: control group), and the presence or absence of mycosis (filled triangle: non-mycotic; empty triangle: mycotic). RPCA shows clustering of samples with similar compositions. Significance was calculated via PERMANOVA (**a**, **b**). Alpha diversity was measured with the Shannon’s diversity index. Significance in alpha diversity was determined by Wilcoxon tests (**c**, **d**). NS = not significant, * *p* ≤ 0.1, ** *p* ≤ 0.05, *** *p* ≤ 0.01.

Clear compositional differences in both the fungal (ITS) and bacterial (16S) intestinal microbiomes were detected along the survival span of all SBWs (Fig. 4a, fungi: R^2^ = 0.147***; Fig. 4b, bacteria: R^2^ = 0.139***). The treatment (indicated by the colour: fungi: R^2^ = 0.077***; bacteria: R^2^ = 0.053**) clearly impacted the composition of the fungal community, particularly between the fungal treatments and the control group. The presence of an external mycosis also shaped the intestinal microbiome, when the diversity of fungi between-samples was considered (indicated by different shape: fungi: R^2^ = 0.077***; bacteria: R^2^ = 0.026**). Considering the fungal microbiome (Fig. 4a), intestinal samples from mycotic SBWs formed a distinct cluster, regardless of the fungal treatment used. However, they were clearly separated from non-mycotic SBWs (specimens from the medium survival category). Such clear separation was not observed in the intestinal bacteria of the SBWs (Fig. 4b), where samples of mycotic and non-mycotic specimens overlapped. Although SBWs with a long survival span exhibited significant variation in fungal diversity, the same samples clustered together when the intestinal bacteria were considered.

The average alpha diversity values (Shannon index) were similar when comparing the fungal diversity with (Fig. 4c) and without *Metarhizium* (Fig. S2) and the patterns remained the same. Within-sample fungal diversity was significantly different when comparing fungal treatments (*M. brunneum* and *M. robertsii*) with the control group (*p* < 0.01). Significant differences in the alpha diversity of intestinal fungi and bacteria (Fig. 4c, d) were observed in relation to the presence or absence of mycosis, as well as when comparing the survival category “short” with “medium” and “long”. It is important to note that SBWs with a short survival time (i.e. death within ten days) were almost exclusively specimens that were treated with *M. robertsii* or *M. brunneum* (15 SBWs, one of them from the control group) and exhibited mycosis. Detailed results of alpha and beta diversity, the Wilcoxon test and PERMANOVA, can be found in Supplementary Tables S1, S2 and S3, respectively.

### Distinct microbes based on survival span and mycosis

**Fig. 5:**
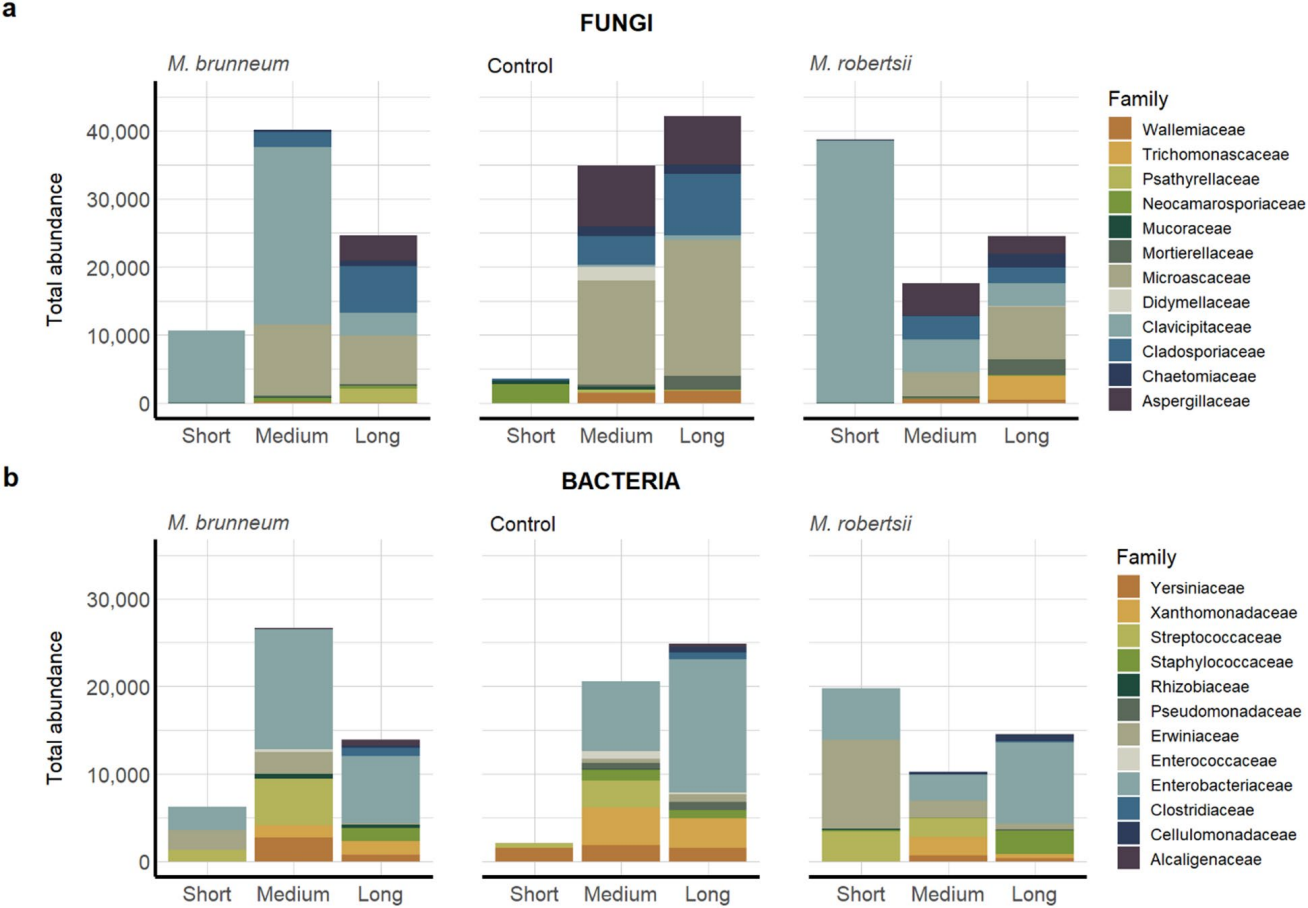
Microbial composition of the SBW intestine. Total abundances of fungal (**a**) and bacterial (**b**) taxa are shown at family-level within the fungal treatments (*M. brunneum*, *M. robertsii*) and the control group, considering the survival span. Intestinal samples were categorized based on the survival of each individual (short: ≤ ten days; medium: ≤ 50 days; long: > 50 days) and the presence (red) and absence (blue) of a mycosis. Low prevalent taxa were filtered with a 10% prevalence threshold and a minimum abundance of 1,000.

In accordance with the results of the alpha diversity analysis, distinct microbial patterns were observed in the intestines of SBWs in the different fungal treatment types (*M. brunneum* and *M. robertsii*) and the control group, considering the survival span of each specimen (Fig. 5). The fungal diversity of the control group remained relatively stable regardless of the survival span, with a dominance of Microsacaceae (Fig. 5a). In contrast, the survival span impacted bacterial diversity, resulting in an increase in the abundance of Enterobacteriaceae in long-lived specimens (Fig. 5b). The only individual in the control group that did not survive more than ten days showed significantly lower diversity, with a dominance of Neocamarosporiaceae and Yersiniaceae. It is obvious that, due to *Metarhizium*-treatment, the family Clavicipitaceae (which includes *Metarhizium*) dominated the intestines of SBWs with a short survival span. Additionally, a dominance of Erwiniaceae was observed specifically in *M. robertsii*-treated individuals (short-lived specimens). Most SBWs treated with *M. brunneum* survived for at least ten days and were dominated by Enterobacteriaceae, as were long-lived SBWs in every treatment group. No dominant fungal taxa were observed in treated SBWs that survived for over 50 days, unlike the control group where Microsacaceae was dominant.

**Fig. 6:**
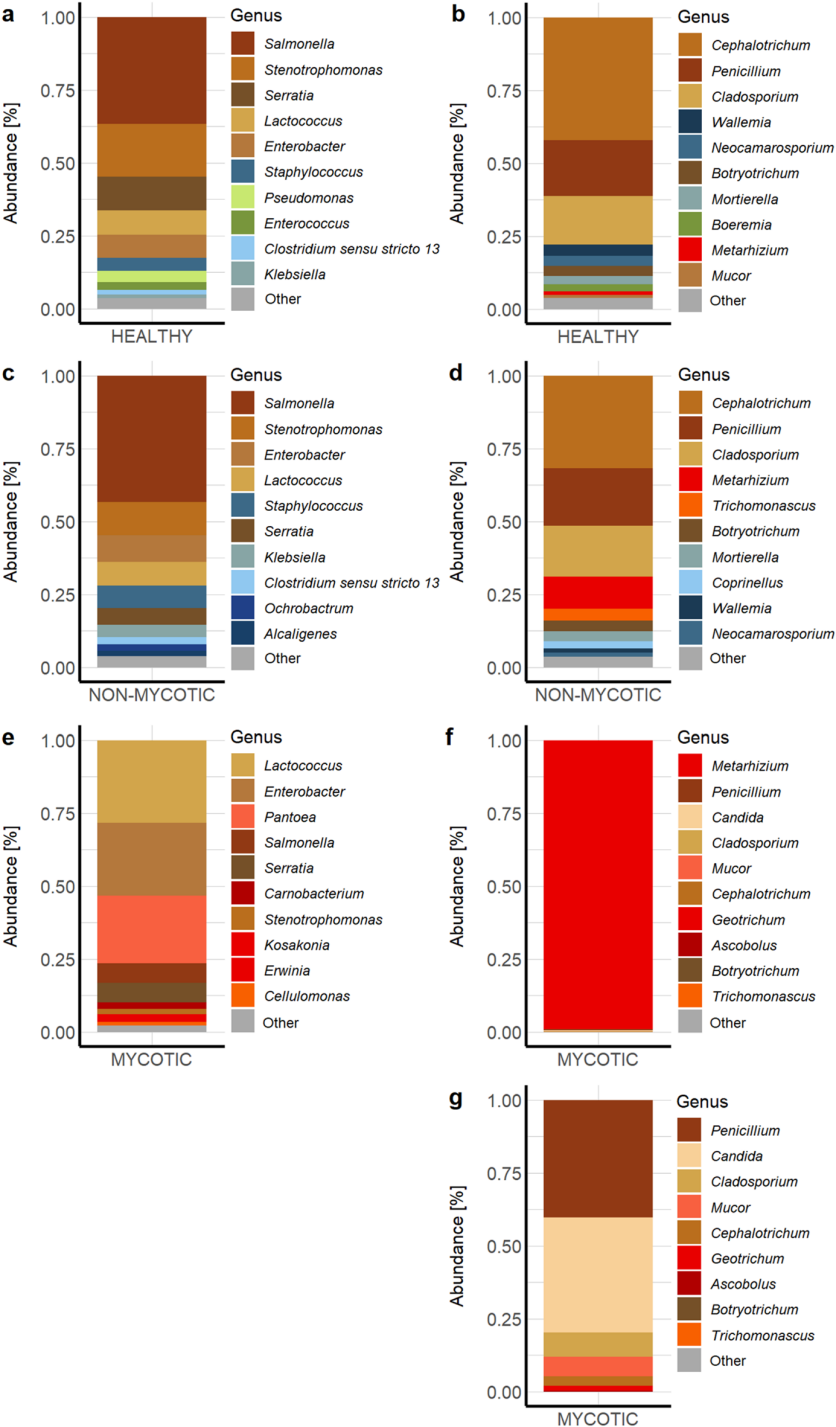
Relative abundances of the ten most abundant genera in the sugar beet weevil’s intestine. The relative abundance of bacterial (**a**,** c**,** e**) and fungal (**b**,** d**,** f**,** g**) taxa is shown at genus-level within the fungal treatments (*M. brunneum*, *M. robertsii*) comparing MYCOTIC and NON-MYCOTIC samples with the control group (HEALTHY). Low abundant fungal and bacterial taxa were grouped into “Other” (coloured in grey). Most abundant fungal taxa of MYCOTIC individuals are detailed without *Metarhizium* (**g**).

Several differences were observed among the ten most abundant taxa in the intestinal microbiome of SBWs based on the presence and absence of an external mycosis. The intestines of healthy SBWs (control group; Fig. 6a and b) comprised high abundances of the fungal genus *Cephalotrichum* (42.1% rel. abundance) (Sordariomycetes: Microascaceae) and the bacterial genus *Salmonella* (36.7%) (Gammaproteobacteria: Enterobacteriaceae). The intestinal microbiome of healthy SBWs also included *Penicillium* (19.1%) (Eurotiomycetes: Aspergillaceae) and *Cladosporium* (16.5%) (Dothideomycetes: Mycosphaerellaceae) as well as *Stenotrophomonas* (18.1%) (Gammaproteobacteria: Xanthomonadaceae) and *Serratia* (among them *S. marcescens*) (11.5%) (Gammaproteobacteria: Yersiniaceae). A similar pattern was observed in the intestine of SBWs treated either with *M. brunneum* or *M. robertsii* but without visible mycosis at the time of death (Fig. 6c and d). Both groups were uniquely enriched by the bacterial genera *Staphylococcus* (Bacilli: Staphylococcaceae) and *Klebsiella* (among them *K. pneumoniae*) (Gammaproteobacteria: Enterobacteriaceae) as well as the fungal genera *Wallemia* (Wallemiomycetes: Wallemiaceae), *Neocamarosporium* (Dothideomycetes: Neocamarosporiaceae) and *Mortierella* (among them *M. wolfii* and *M. elongata*) (Mortierellomycetes: Mortierellaceae) when compared to mycotic SBWs. Differences between the intestinal microbiome of healthy and non-mycotic SBWs included the presence of *Pseudomonas* (Gammaproteobacteria: Pseudomonadaceae) and *Enterococcus* (among them *E. faecalis*) (Bacilli: Enterococcaceae) in the control group, while *Alcaligenes* (among them *A. faecalis*) (Betaproteobacteria: Alcaligenaceae) and *Coprinellus* (Agaricomycetes: Psathyrellaceae) uniquely enriched non-mycotic individuals. Although the control group was not treated, *Metarhizium* was one of the ten most abundant taxa with about 1.3% relative abundance. In non-mycotic SBWs, *Metarhizium* (10.9%) was the fourth most abundant fungal taxon.

Due to the fungal treatments, *Metarhizium* dominated the intestine of mycotic weevils (Fig. 6f) (99.1% rel. abundance). Although at low abundance, *Penicillium* (among them *P. expansum*) and *Candida* (Saccharomycetes: incertae sedis) were the next most abundant fungal taxa in mycotic specimens (Fig. 6g). Next to the fungal genus *Candida*, also *Geotrichum* (*G. silvicola*) (Saccharomycetes: Dipodascaceae) and *Ascobolus* (Pezizomycetes: Ascobolaceae) were uniquely present in the ten most abundant fungal taxa in the intestines of mycotic SBWs. Additionally, these intestinal samples were enriched by *Lactococcus* (28.3%) (Bacilli: Streptococcaceae), *Enterobacter* (24.9%) (among them *E. cloacae*) (Gammaproteobacteria: Enterobacteriaceae) and *Pantoea* (23.2%) (among them *P. agglomerans*) (Gammaproteobacteria: Erwiniaceae) (Fig. 6e). Moreover, the abundance of *Salmonella* (6.7%) and *Stenotrophomonas* (1.8%) decreased drastically in these intestinal samples. In addition, *Carnobacterium* (Bacilli: Carnobacteriaceae), *Erwinia* and *Kosakonia*, (Gammaproteobacteria: Enterobacteriaceae) as well as *Cellulomonas* (Actinomycetes: Cellulomonadaceae) were one of the most abundant bacterial genera.

### Intestinal indicator taxa associated with *Metarhizium*-mycosis

**Fig. 7:**
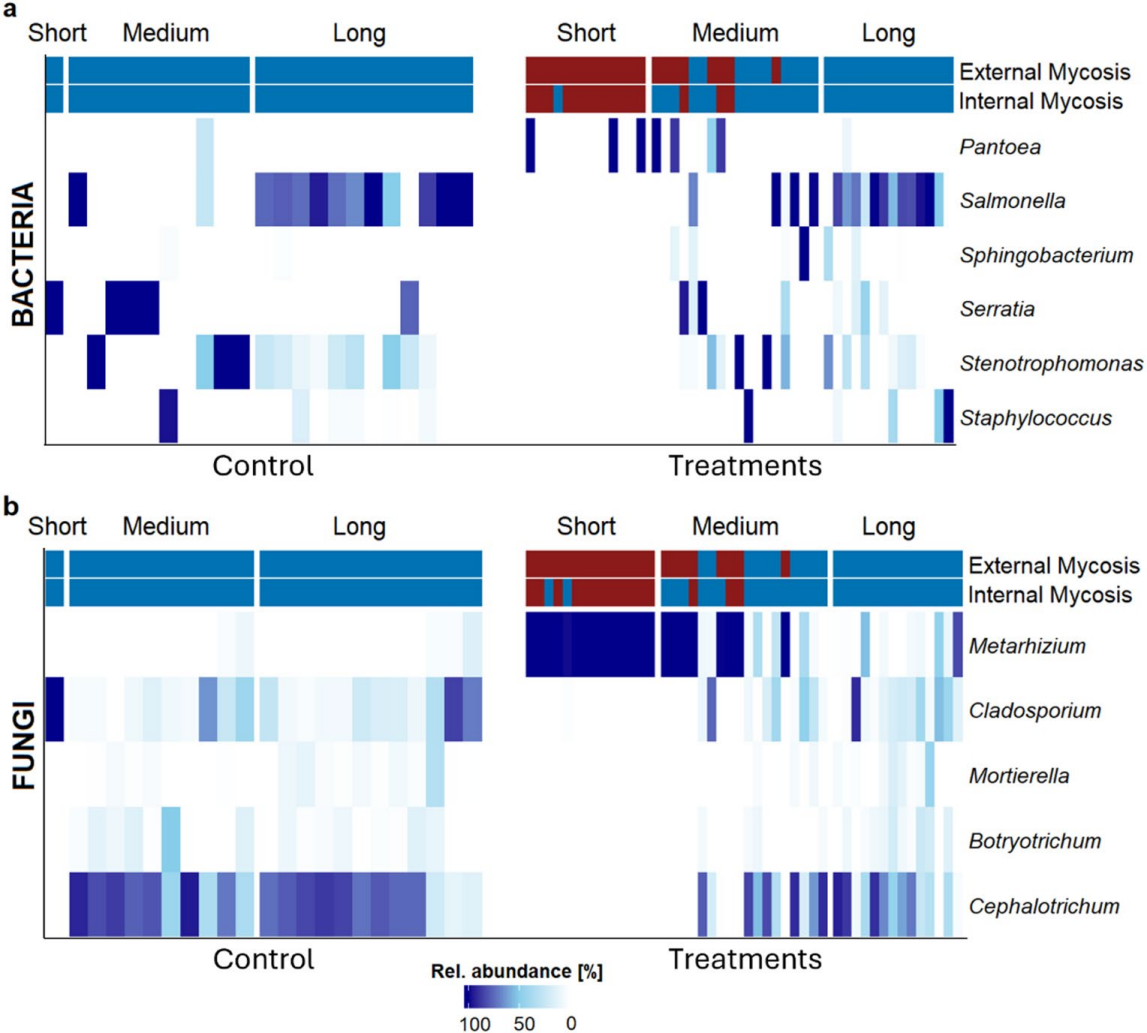
Indicator taxa analysis. Bacterial (**a**) and fungal (**b**) indicator taxa in the intestine of SBWs treated with *M. brunneum* and *M. robertsii* (excluding the control group). DESeq2 analysis was done at genus level, considering only samples with a prevalence of 10%. Significant taxa (BH-adjusted *p* < 0.05; log2FC coefficients > |2|) indicating the presence or absence of an external or internal mycosis are stated. Relative abundances [%] of each indicator taxon in each sample were visualized in heat maps for the treated SBWs and the control group, considering the survival span of each weevil (short: ≤ ten days; medium: ≤ 50 days; long: > 50 days) and presence or absence of a mycosis. Low relative abundances (around 0% of total reads in a sample) were coloured in a white spectrum, great relative abundances were coloured in a blue spectrum (up to 100% of total reads in a sample).

Differential abundance analysis revealed indicator taxa being associated with both, presence and absence of external and internal mycosis after fungal treatment. The most non-mycotic associated taxa were the fungal genera *Cephalotrichum* and *Botryotrichum*, and the bacterial genera *Staphylococcus*, *Stenotrophomonas*, *Sphingobacterium* and *Serratia* (log2FC coefficients > 10). According to their relative abundance per sample (Fig. 7b), *Cephalotrichum* and *Botryotrichum* were enriched in almost every non-mycotic weevil regardless of survival span, and in each specimen of the control group, except for the individual of the survival category “short” (*n* = 1). The fungal genus *Mortierella* mostly present in SBWs with a longer survival span in both, the treated (non-mycotic) and control group. Moreover, the bacterial genus *Salmonella* was also highly abundant in non-mycotic SBWs with a longer survival span (Fig. 7a). Similarly, *Salmonella* enriched the intestine of weevils in the control group surviving more than 50 days. A log2FC coefficient below 10 was determined for both species in relation to external mycosis, while a coefficient above 10 was determined for internal mycosis (absent). Interestingly, *Cladosporium* and *Serratia* dominated the only individual of the control group that died within the first ten days in the experiment. In comparison to non-mycotic SBWs, only two indicator taxa were identified in the intestine of SBWs correlating with the presence of mycosis. Compared to non-mycotic SBWs, *Metarhizium* was the only fungal indicator taxon associated with the presence of both external and internal mycosis, with log2FC coefficients of -6 and − 9, respectively. *Metarhizium* was present at high abundance in the intestines of SBWs that had been successfully infected (no other indicator taxa were observed), and at relatively low abundance in individuals with a long survival span and higher abundances of other indicator taxa. The bacterial genus *Pantoea* was strongly associated with both the presence of an external mycosis and the absence of internal mycosis. Associations of other indicator taxa were unaffected by the localization of mycosis in SBWs. This bacterial genus was highly abundant in the intestines of some SBWs affected by mycosis caused by both species. The log2FC coefficients can be found in the Supplementary File S1, Tab. S4.

The intestinal microbiome of SBWs was found to differ according to sex of individuals without (Supplementary File S1, Fig. S3a, c) or with mycosis (Supplementary File S1, Fig. S3b, d). According to the phylogenetic heat tree analysis, the intestinal microbiome of both sexes of mycotic SBWs was characterised by low microbial diversity. While the bacterial genus *Enterobacter* dominated male SBWs regardless of the mycosis status (Supplementary File S1, Fig. S3c, d), *Metarhizium* appeared to tend to enrich female specimens slightly more than male ones (Supplementary File S1,Fig. S3a, b). Non-mycotic male SBWs were additionally enriched in several fungal genera and species, including *Metarhizium*, *C. stemonitis*, *P. expansum* and *Cladosporium*. By contrast, female SBWs showed greater enrichment by multiple bacterial genera including *Salmonella* and *Stenotrophomonas*, the bacterial phylum Firmicutes as well as the fungal genera *Mortierella* and *Botryotrichum*.

### *M. brunneum* vs. *M. robertsii*: distinct taxonomic patterns in mycotic and non-mycotic SBWs

**Fig. 8:**
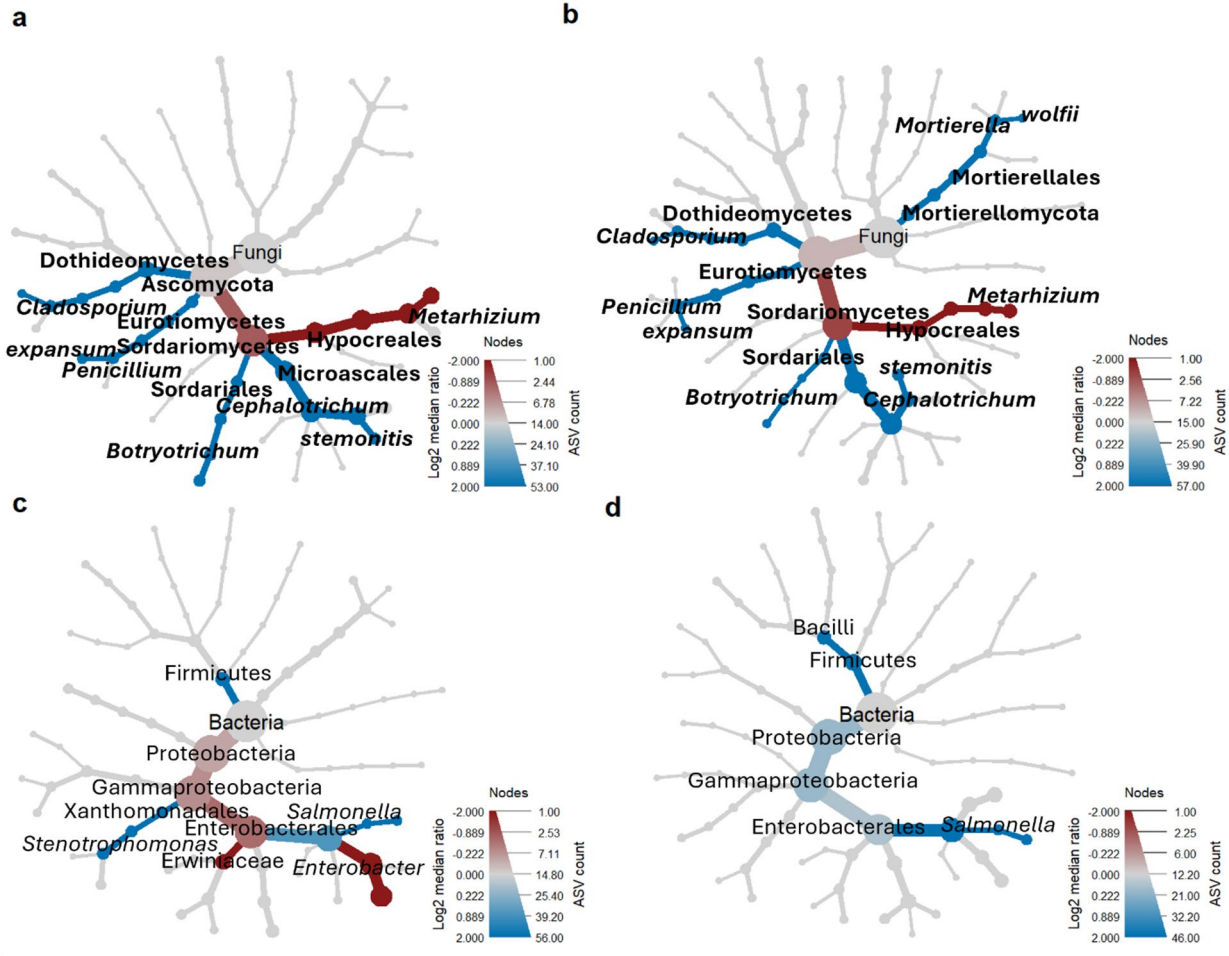
Differences in microbial composition in mycotic and non-mycotic SBWs. Fungal (**a**,** b**) and bacterial (**c**,** d**) composition in the intestine of weevils treated with *M. brunneum* (**a**, **c**) and *M. robertsii* (**b**, **d**). Different taxonomic ranks are shown in nodes, starting from the highest rank (largest nodes; great numbers of ASVs assigned to the taxon) to the genus or species level at the end of the branches. Coloured nodes, based on the log2 median ratio proportion [-2;2], represent taxa which are more enriched in mycotic (red) or non-mycotic (blue) samples. Significant taxa (Wilcoxon *p* < 0.05) are highlighted.

Phylogenetic heat tree analysis revealed significant differences in the microbiome of SBWs’ intestines, considering the presence and absence of external mycosis either caused by *M. brunneum* (Fig. 8a, c) or *M. robertsii* (Fig. 8b, d). These differences were observed in both, the fungal (Fig. 8a, b) and bacterial microbiomes (Fig. 8c, d). Non-mycotic SBWs, either treated with *M. brunneum* or *M. robertsii*, were enriched by the phylum Firmicutes and Enterobacteriaceae including the genus *Salmonella*. Additionally, multiple fungal species were present in intestines of non-mycotic weevils including the genera *Cladosporium* (Dothideomycetes), *Botryotrichum* (Sordariales) and species like *P. expansum* (Eurotiomycetes) and *Cephalotrichum stemonitis* (Microascales). *Mortierella wolfii* (Mortierellomycota) was also found to be significantly more abundant in non-mycotic specimens, but only when they had been treated with *M. robertsii*. In contrast, trends to distinct bacterial patterns were observed in the intestines of non-mycotic SBWs when comparing the two fungal treatments. *Stenotrophomonas* (Xanthomonadales) were present in the intestines of non-mycotic weevils when treated with *M. brunneum*. Moreover, the bacterial family Erwiniaceae (Enterobacterales) and the genus *Enterobacter* were more abundant in mycotic than non-mycotic SBWs when treated with *M. brunneum*. Similar results were revealed by MaAsLin2, with a mycosis caused by *M. brunneum* being associated with multiple bacterial species. The bacterial genus *Serratia* and the fungal species *C. stemonitis* were the most significant taxa associated with the absence of mycosis. This analysis did again not determine any bacterial taxa to be associated with mycosis caused by *M. robertsii*. Only the fungal species *P. expansum* significantly correlated with the absence of mycosis (Supplementary File S1, Tab. S5).

## Discussion

The combined effects of climate change, the prohibition of plant protection products^6,9,53^ and the absence of effective alternatives have resulted in frequent SBW outbreaks and continue to present a significant challenge to sugar beet cultivation. Entomopathogens, including fungi and bacteria, cause infection and death in insects^54^ and are thus being used as biological control agents. The entomopathogenic genus *Metarhizium* has already demonstrated its effectiveness in controlling various pests^13,16^, including the SBW^15^, and has also been shown to affect a host’s microbiome^22,23^. Insects harbour a variety of microbial species that are essential for the survival of their hosts, but some of these species may also become virulent towards their host^20^. A comprehensive understanding of the dynamics of the intestinal microbes in infected insect pests is crucial for harnessing these benefits effectively. It is already described that changes in intestinal microbial composition affect insect metabolism and physiology^55^. This may also be the case for *Metarhizium*-infected SBWs. Therefore, in this study, we investigated the microbial differences in the intestines of untreated and *Metarhizium*-treated SBWs. We identified key microbial indicators of healthy intestinal microbiomes, as well as potential opponents of *Metarhizium* and agonistic microbial species.

We acknowledge the potential limitations of this study, given that only 70 SBWs were analysed, collected from three locations in the same year. To gain a more comprehensive understanding, a multi-year and multi-site study would be optimal. Since the larval stage cannot only develop on sugar beet roots, but also on the roots of various weeds (e.g. *Atriplex patula*, *Chenopodium album*)^56^ and *Metarhizium* spp. are soil-borne fungi^57^, the impact of *Metarhizium*-treatments on the intestinal microbiome of the larvae should also be considered in future research. Moreover, fungal colonization of the rhizosphere and the endophytic colonization of other plant parts not only promote plant growth but also enhance resistance against phytopathogens^10,22^. Given these beneficial effects, it is of great interest to assess the overall health of *Metarhizium*-treated plants, including their sucrose content, and the presence of plant pathogens and pests. This would allow us to gain a comprehensive understanding of the microbial interactions, as well as their impact on the SBW and sugar beet. Such tri-trophic interactions are complex dynamics in which each component influences and is influenced by the others and by external factors^58^. These interactions range from plant-specific microbes that can manipulate insect behaviour or promote plant health and tolerance, to beneficial intestinal microbiomes that outcompete harmful microbes. Moreover, not only the intestinal but also cuticular microbiome of the SBW might harbor important defense-related microbes^55^. Despite these limitations, we emphasize the value of our research as basis for future studies.

### A diverse intestinal microbiome protects SBW from *Metarhizium*

Healthy SBWs (control group and non-mycotic individuals) differed significantly in microbial diversity and composition from mycotic ones. Intestinal microbes are known to influence the immune system, nutrient processing and development of an insect as a form of mutualism or symbiosis^20,59^, potentially preventing insect diseases. In our study, a significant proportion (75–80%) of treated SBWs resisted *Metarhizium*-infection and harboured diverse intestinal microbiomes similar to the control group. The intestinal microbiome of both, healthy and non-mycotic SBWs was enriched by several microbes, including *Stenotrophomonas*, *Staphylococcus* and *Pseudomonas* and *Mortierella.*

*Stenotrophomonas*^14^ and *Staphylococcus*^60^ can degrade plant cell wall components. Most herbivorous insects harbour intestinal bacteria that facilitate the degradation of cellulose and other plant fibres^20^. The ability to acquire nutrients more easily can strengthen an insect and thus indirectly provide an advantage when it comes to defending itself against fungal attacks. Moreover, *Staphylococcus* and *Pseudomonas* produce siderophores that inhibit entomopathogens in bark beetles (*Ips typographus*)^61^, suggesting similar protective roles in SBWs. The fungal class Mortierellomycetes and various *Mortierella* spp. have been observed as part of the sugar beet microbiome^30^. The genus is known to antagonize phytopathogenic fungi^62^ and plant-parasitic nematodes^63^, thereby contributing to maintain a plant’s health. However, its function within the insect intestine has not yet been described. Since *Pseudomonas* has already shown to exhibit antifungal activity against both phytopathogens and entomopathogens^61,64^, similar observations could be made in future research on *Mortierella*. Although the exact function of each species remains to be absolutely clarified; our results, including a highly diverse intestinal microbiome, align with the fact that species-rich communities provide various microbiota-host interactions and are therefore more resistant to invasions^20^. Since microbial diversity is often considered as a biomarker for healthy microbiomes, not only for plants^65^, but also in humans^66^ or other organisms and environments, such as soil^67^, manipulating the intestinal microbiome in reducing its diversity (e.g. by applying antagonists to host plants) may be a key factor for improved biological pest control.

As a matter of fact, successful *Metarhizium*-mycosis was associated with species-poor fungal communities in SBWs. This is consistent with previous findings that increased susceptibility and disease incidence are connected with species-poor microbial systems^20,65^. Hummadi *et al*. (2022) also noted eukaryotic microbes as the main competitors to *M. brunneum*^68^. However, it remains unclear whether mycosis caused the decline in diversity, or reduced diversity enabled the infection in the first place. The former can be explained by the fact that entomopathogenic fungi, specifically *M. brunneum*, can produce a wide range of antimicrobial VOCs, which inhibit bacteria, yeasts and filamentous fungi. The activity depends on the fungal developmental stage, cultural conditions, and synergistic effects^68^. In support of the latter, high variability of microbial diversity deems a natural phenomenon attributed to diet and environment in the spruce budworm’s intestine (*Choristoneura fumiferana*)^69^, whereas a high microbial diversity is considered as a biomarker for healthy microbiomes^65^. Moreover, Kabaluk, Li-Leger and Nam (2017) connected increased wireworm mortality caused by *M. brunneum* with reduced environmental bacteria^23^. In our study, bacterial communities remained relatively stable across treatments and were more affected by the presence of mycosis than by *Metarhizium*-exposure alone.

Notably, *Pantoea* was strongly associated with the presence of external mycosis but also with the absence of internal mycosis. *P. agglomerans* known to antagonise *M. brunneum* in wireworms via antimicrobial compounds^23^ may have prevented intestinal mycosis in some individuals. In fact, the genus itself, including *P. agglomerans*, mainly reside in the intestines of insects^70^ and have been observed to be relatively unaffected by VOCs produced by *M. brunneum*^68^. The successful prevention of intestinal mycosis (internal) in this study could therefore be explained by *Pantoea*’s intestinal location and the fact that *Metarhizium* invades through the cuticle before reaching internal organs^71^. An external mycosis might be already successful and lethal before VOCs even reached *Pantoea* in the intestine, whereas VOCs must make direct contact with the target^68^. On the other hand, *P. agglomerans* also produces VOCs itself, which are important for suppressing and communicating with other microorganisms^72^. Thus, while *Pantoea* may provide protective behaviour very localized in the intestine, *Pantoea* as a single intestinal antagonist cannot fully prevent lethal mycosis.

Similar to the findings of Kabaluk, Li-Leger and Nam (2017)^23^, we can conclude that suitable conditions in entomopathogenic attacks include, among other factors, a reduced diversity associated with a susceptible host. Since *Pantoea* was mainly observed in mycotic SBWs, we broaden these findings by assuming that antagonistic microbes are not triggered into proliferation unless certain host conditions or physiological changes have occurred. Indeed, the production of VOCs by entomopathogens at the cuticle adhesion stage can induce behavioural or physiological responses in insects, thereby influencing their immune response^19^. Considering that an insect’s intestinal microbiome is also part of its immune system^20^, we hypothesize that certain fungal VOCs trigger the SBW’s immune system and indirectly activated *Pantoea*-growth when SBWs were attacked by *Metarhizium*. However, this study has not involved an analysis of emitted VOCs; thus, we can only suggest that a certain combination of VOCs, host conditions and target microbe stimulate the proliferation of antagonistic bacteria. Nevertheless, a diverse intestinal microbiome, rather than a single *Metarhizium*-antagonist, successfully prevented lethal mycosis.

### Insecticidal species in the intestinal microbiome of the SBW

The broad ecological roles of *Metarhizium* spp. make them keystone organisms in sustainable agricultural systems^73^. Diverse ecosystems comprise various species that not only regulate insect pests, suppress plant pathogens and parasitic nematodes, but also act as biofertilizers, providing direct and indirect biological control and contributing to plant health and development^10,54^. While cooperative and competitive interactions shape microbial networks and symbiotic relationships with a host plant^24^, the effects of applying *Metarhizium* in combination with other microbes are not yet known. In our study, mycotic SBWs harboured species-poor fungal communities, whereas non-mycotic individuals were characterized with diverse microbiomes comprising not only insect-beneficial species but also potentially pathogenic bacteria and fungi. The insect intestinal microbiome can have synergistic or antagonistic effects depending on an entomopathogenic species and its host. Especially intestinal bacteria can become virulent depending on physiological or environmental conditions as well as a disruption of the normal microbial composition^18^. It can therefore be suggested that certain microbial species may have engaged in insect-symbiotic behaviour or activated their virulent traits in response to *Metarhizium*-infection. Although mycosis was not the cause of death in all treated individuals, the presence of *Metarhizium* might have increased the insect-susceptibility against other species with insecticidal traits.

As a matter of fact, *Cladosporium* and *Penicillium* were observed in the intestines of all SBWs and additionally identified as indicator taxa in intestines of non-mycotic individuals. Although some species of *Penicillium* might have symbiotic connections with their insect host^74^, *P. expansum* has been reported to negatively affect the development and viability of *Drosophila* larvae^75^. Anti-insect properties of both *Penicillium* and *Cladosporium* include the production of insecticidal secondary metabolites^74,76^. *Cladosporium* was, next to the bacterial genus *Serratia*, predominantly observed in the intestine of the only SBW in the control group to die within the first ten days, potentially contributing to its rapid death.

Since fungi can interact with various bacterial species during the development of mycosis in insects^18^, it is reasonable to identify associated taxa, even in the absence of visible mycosis. *A. faecalis* and *Salmonella* were enriched in non-mycotic individuals, with the former being described as colonizing various nematodes and successfully killing insects^77^. As a pathogenic intestinal bacterium, *A. faecalis* has the potential to be used as a biological control agent in the management of insect pests^18^. *Salmonella* (Enterobacteriaceae) was present in long-lived treated SBWs and in the control group. Previous studies have shown an increase in Enterobacteriaceae in larvae of the Colorado potato beetle after *M. robertsii*-infection, which contributes to subsequent decomposition of the host and release of inorganic nitrogen^22^. Given the fact that *Metarhizium* also supplies its host plant with insect-derived nitrogen^78^ suggests not only insect-lethality but also plant growth promotion by both *Metarhizium* and members of Enterobacteriaceae such as *Salmonella* and *Enterobacter*. The latter genus was observed in all SBWs, but especially in mycotic individuals after *M. brunneum* infection.

*Serratia* (Yersiniaceae) was strongly associated with the absence of mycosis, especially when attacked by *M. brunneum*. In fact, the family Yersiniaceae and the genus itself were one of the few taxa present in the only SBW of the control group that died within the first ten days of the experiment. Among the identified species was *S. marcescens*, a well-known insect-pathogen, which can produce hydrolytic enzymes (e.g. proteases and a chitinase), some of which have been shown to be insect toxins^79,80^. Multiple strains have already been tested for their pathogenicity against insects, including beetles, butterflies and flies^81^. The genus itself is omnipresent in nature and is not only known as an insect-pathogen but also exhibits plant growth promoting traits^82^. Fungal infections caused by *M. robertsii* have been shown to significantly increase the abundance of *Serratia* with an effect on mycosis in Colorado potato beetles^22^. Thus, in terms of pest management and plant protection, *Serratia* not only offers the benefit of targeted SBW control, but also the potential to improve sugar beet plant health. Considering that the entomopathogens *M. brunneum* and *M. robertsii* also promote plant health and growth^16,68^ suggests a synergistic effect when *Serratia* is applied in combination, or subsequently. However, it has also been reported that *E. cloacae*, a species of the above-mentioned genus *Enterobacter*, can reduce the effectiveness of pest control by *M. anisopliae* and *S. marcescens*^60^. Although various entomopathogens have the potential to increase mortality in SBWs, their diverse intestinal microbiome may reduce the efficacy of mycosis and other insecticidal effects. Further research, including feeding or injection experiments with specific strains, is required to confirm the potential of synergistic interactions between *Metarhizium* and *Serratia* against insect-beneficial species, and to ultimately improve biological control against SBWs.

### Towards sex-specific pest management of the SBW

Although the majority of the genome is identical in both sexes, sexual dimorphism is ubiquitous in the animal kingdom, manifesting itself in different physiology, morphology, and behavior^83,84^. Such differences have also already been described for the SBW^4^, thus, sex-dependent reactions to entomopathogenic attacks seem plausible. In fact, male *Drosophila* individuals seem to be more affected by fungal infections than female individuals^85^. A similar pattern was also observed in this study: male SBWs lived generally shorter and had a higher risk of death from mycosis compared to female individuals, despite the slightly higher abundance of *Metarhizium* in females.

The increased risk of death by mycosis was accompanied by the bacterial family Erwiniaceae and the bacterial genus *Enterobacter*. The latter was also part of the intestinal microbiome of male individuals that survived an entomopathogenic attack (non-mycotic). According to the ability of *E. cloacae* to reduce the effectiveness of pest control by *M. anisopliae*^60^, an immune reaction, unsuccessful in mycotic and successful in mycotic individuals, might have triggered the proliferation of such proposed insect-beneficial species. The presence of the already mentioned fungal genera *Cladosporium* and *Penicillium* and (next to beneficial traits of some species) the insecticidal features^74–76^, might also have contributed to higher death rates in male SBWs. According to the (partially significant) longer survival span of female SBWs (in comparison to male individuals), the intestinal microbiome was enriched by *Salmonella* contributing to the decomposition^22^ of such long-living individuals. Another noteworthy difference was the abundance of the proposed insect-beneficial bacterial genus *Stenotrophomonas*^14^ and the fungal genus *Mortierella*. Although, its specific feature in insect intestines is not yet know, an advantage in survival can be hypothesised. Female SBWs exhibit increased leaf consumption during maturation feeding^56^, whereby *Stenotrophomonas* could be beneficial for digestion. In addition, *Mortierella* has been identified as part of the sugar beet microbiome^30^, which makes it plausible that such microbes beneficial to insects accumulate in female SBWs at a higher rate, thereby mitigating the harmful effects of pathogen attacks, e.g., by *Metarhizium*.

In nature, male SBWs were observed to leave their winter habitat and start maturation feeding earlier than females, a form of sexual dimorphism known as protandry^4,86^. In this scenario, males gain a fitness advantage and increased mating success^87,88^. This behaviour, combined with their increased susceptibility to mycosis and the higher risk of death likely linked to their intestinal microbiome, could form the basis for a sex-based pest management strategy. Recently, a reduced reproductive potential was observed in male *D. melanogaster* during incubation period following *M. brunneum* infection^89^. Thus, an early application of *M. robertsii* with its fast mortality rate (compared to *M. brunneum*) targeting male SBWs in combination with pheromone-based mass traps^90^ might accomplish an unbalanced female: male ratio that ultimately could lead to reduced offspring. Koschier, Dittmann and Spangl (2024) have shown that compared to female SBWs, male SBWs responded positively, next to young sugar beet and chard plants, to the odour of the garden orache^91^. Altogether, by leveraging sexual dimorphism and protandry, an early spring application of male-specific insecticidal microbes following *M. robertsii* could substantially enhance the efficacy of *Metarhizium*-based biological control. Integrating this strategy with male-targeted pheromone mass-traps may further improve pest management against the sugar beet weevil.

## Conclusion

The intestinal microbiome of insects possesses various functions including immune response. Infection by the entomopathogenic fungus *Metarhizium* can affect insect’s intestinal microbes. It appears that species-poor intestinal communities are more susceptible to successful mycosis. It is possible that the resistance of SBWs to *Metarhizium*-infections might be mediated by a diverse intestinal microbiome harbouring insect-beneficial microbes such as *Pantoea agglomerans* and *Enterobacter*. Certain species of the fungal genera *Cladosporium* and *Penicillium*, and *Serratia marcescens* possess insecticidal features, potentially contributing to a more rapid SBW death and increased mortality. Ultimately, the efficacy of biological control depends on various factors, including microbe-microbe and host-microbe interactions. Notably, extending the concept of sexual dimorphism in insects to include intestinal microbiome patterns in response to fungal infestation adds an additional dimension to pest control. For an effective biocontrol we therefore emphasise the importance of early application of insecticidal microbes, either in combination with the entomopathogen *Metarhizium* or in a subsequent treatment, primarily targeting male sugar beet weevils.

## Supplementary Information

Below is the link to the electronic supplementary material.

Supplementary Material 1

## Data Availability

The sequence data is available at NCBI and can be accessed with the BioProject accession number PRJNA1330021 (https://www.ncbi.nlm.nih.gov/bioproject/PRJNA1330021).
