# Supplementary material for: Intestinal microbiome interactions influence Metarhizium-based biocontrol efficacy against the sugar beet weevil
Source: Sci Rep. 2026 Jan 13;16:5174. doi: 10.1038/s41598-026-36038-8 (PMC12881356; doi:10.1038/s41598-026-36038-8)
Supplement: Supplementary file 1 — Supplementary Material 1 [file 41598_2026_36038_MOESM1_ESM.docx]

**Supplementary Table S1** Alpha diversity (Shannon index) values of fungal ITS and bacterial 16S data.

| **Intestinal samples** | **Sex** | **Treatment** | **Location** | **Days until  death** | **Survival  group** | **Internal mycosis** | **External mycosis** | **Shannon index  ITS with  *Metarhizium* spp.** | **Shannon index  ITS without  *Metarhizium* spp.** | **Shannon index  16S** |
| --- | --- | --- | --- | --- | --- | --- | --- | --- | --- | --- |
| ASP_01 | F | *M. brunneum* | Obermallebarn | 35 | Medium | Absent | Absent | 2.015 | 1.977 | 0.997 |
| ASP_02 | F | *M. brunneum* | Obermallebarn | 5 | Short | Present | Present | 0.027 | 0.000 | 0.905 |
| ASP_03 | M | *M. brunneum* | Obermallebarn | 25 | Medium | Absent | Present | 0.087 | 1.060 | 1.587 |
| ASP_04 | F | *M. brunneum* | Obermallebarn | 95 | Long | Absent | Absent | 1.414 | 1.229 | 1.131 |
| ASP_05 | F | *M. brunneum* | Obermallebarn | 35 | Medium | Present | Present | 0.000 | 0.000 | 1.361 |
| ASP_06 | F | *M. brunneum* | Obermallebarn | 94 | Long | Absent | Absent | 0.839 | 0.274 | 1.318 |
| ASP_07 | M | *M. brunneum* | Obermallebarn | 17 | Medium | Absent | Present | 0.127 | 0.000 | 1.366 |
| ASP_08 | F | *M. brunneum* | Obermallebarn | 9 | Short | Present | Present | 0.038 | 0.000 | 0.928 |
| ASP_09 | M | *M. brunneum* | Rückersdorf | 13 | Medium | Absent | Present | 0.000 | 0.000 | 0.852 |
| ASP_10 | M | *M. brunneum* | Rückersdorf | 45 | Medium | Absent | Absent | 1.290 | 0.979 | 1.135 |
| ASP_11 | F | *M. brunneum* | Rückersdorf | 18 | Medium | Absent | Present | 0.000 | 0.000 | 1.640 |
| ASP_12 | F | *M. brunneum* | Rückersdorf | 46 | Medium | Absent | Absent | 1.591 | 1.591 | 0.917 |
| ASP_13 | M | *M. brunneum* | Rückersdorf | 63 | Long | Absent | Absent | 1.330 | 1.300 | 2.207 |
| ASP_14 | F | *M. brunneum* | Rückersdorf | 74 | Long | Absent | Absent | 1.418 | 1.347 | 1.887 |
| ASP_16 | F | *M. brunneum* | Rückersdorf | 38 | Medium | Absent | Absent | 1.206 | 1.206 | 1.479 |
| ASP_17 | M | *M. brunneum* | Ruppersthal | 42 | Medium | Absent | Absent | 1.614 | 1.614 | 1.677 |
| ASP_18 | F | *M. brunneum* | Ruppersthal | 64 | Long | Absent | Absent | 0.418 | 0.418 | 1.629 |
| ASP_19 | F | *M. brunneum* | Ruppersthal | 70 | Long | Absent | Absent | 0.904 | 0.904 | 2.059 |
| ASP_20 | M | *M. brunneum* | Ruppersthal | 6 | Short | Present | Present | 0.000 | 0.000 | 0.573 |
| ASP_21 | F | *M. brunneum* | Ruppersthal | 23 | Medium | Absent | Absent | 1.807 | 1.697 | 2.457 |
| ASP_22 | F | *M. brunneum* | Ruppersthal | 66 | Long | Absent | Absent | 1.500 | 1.353 | 2.302 |
| ASP_23 | M | *M. brunneum* | Ruppersthal | 22 | Medium | Present | Present | 0.000 | 0.000 | 0.768 |
| ASP_24 | F | *M. brunneum* | Ruppersthal | 42 | Medium | Absent | Present | 0.000 | 0.000 | 0.909 |
| ASP_25 | M | Control | Obermallebarn | 8 | Short | Absent | Absent | 0.824 | 0.824 | 1.082 |
| ASP_26 | F | Control | Obermallebarn | 92 | Long | Absent | Absent | 0.911 | 0.576 | 1.190 |
| ASP_27 | F | Control | Obermallebarn | 71 | Long | Absent | Absent | 1.298 | 1.297 | 1.101 |
| ASP_28 | F | Control | Obermallebarn | 56 | Long | Absent | Absent | 1.281 | 1.149 | 1.445 |

**Supplementary Table S1** Alpha diversity (Shannon index) values of fungal ITS and bacterial 16S data.

| **Intestinal samples** | | **Sex** | **Treatment** | **Location** | **Days until  death** | **Survival  group** | **Internal mycosis** | **External mycosis** | **Shannon index  ITS with  *Metarhizium* spp.** | **Shannon index  ITS without  *Metarhizium* spp.** | **Shannon index  16S** |
| --- | --- | --- | --- | --- | --- | --- | --- | --- | --- | --- | --- |
| ASP_29 | F | Control | Obermallebarn | 32 | Medium | Absent | Absent | 1.754 | 1.754 | 1.066 |  |
| ASP_30 | F | Control | Obermallebarn | 63 | Long | Absent | Absent | 1.779 | 1.779 | 1.391 |  |
| ASP_31 | F | Control | Obermallebarn | 32 | Medium | Absent | Absent | 1.641 | 1.641 | 1.053 |  |
| ASP_32 | F | Control | Obermallebarn | 35 | Medium | Absent | Absent | 1.659 | 1.659 | 1.284 |  |
| ASP_33 | F | Control | Rückersdorf | 65 | Long | Absent | Absent | 1.586 | 1.586 | 1.344 |  |
| ASP_34 | F | Control | Rückersdorf | 66 | Long | Absent | Absent | 1.883 | 1.883 | 1.422 |  |
| ASP_35 | F | Control | Rückersdorf | 58 | Long | Absent | Absent | 1.590 | 1.591 | 1.751 |  |
| ASP_36 | F | Control | Rückersdorf | 60 | Long | Absent | Absent | 1.608 | 1.608 | 1.320 |  |
| ASP_37 | F | Control | Rückersdorf | 42 | Medium | Absent | Absent | 1.424 | 1.390 | 1.774 |  |
| ASP_38 | F | Control | Rückersdorf | 45 | Medium | Absent | Absent | 1.756 | 1.688 | 1.170 |  |
| ASP_39 | M | Control | Rückersdorf | 45 | Medium | Absent | Absent | 1.647 | 1.507 | 1.152 |  |
| ASP_40 | F | Control | Ruppersthal | 25 | Medium | Absent | Absent | 1.052 | 1.047 | 1.298 |  |
| ASP_41 | F | Control | Ruppersthal | 87 | Long | Absent | Absent | 0.602 | 0.496 | 0.984 |  |
| ASP_42 | F | Control | Ruppersthal | 66 | Long | Absent | Absent | 1.865 | 1.865 | 1.659 |  |
| ASP_43 | F | Control | Ruppersthal | 65 | Long | Absent | Absent | 1.410 | 1.410 | 1.250 |  |
| ASP_44 | F | Control | Ruppersthal | 36 | Medium | Absent | Absent | 1.368 | 1.368 | 1.414 |  |
| ASP_45 | F | Control | Ruppersthal | 30 | Medium | Absent | Absent | 1.745 | 1.729 | 1.310 |  |
| ASP_46 | M | Control | Ruppersthal | 38 | Medium | Absent | Absent | 1.119 | 1.119 | 0.671 |  |
| ASP_47 | F | Control | Ruppersthal | 71 | Long | Absent | Absent | 1.806 | 1.723 | 1.425 |  |
| ASP_48 | F | *M. robertsii* | Obermallebarn | 73 | Long | Absent | Absent | 1.192 | 1.192 | 1.447 |  |
| ASP_49 | F | *M. robertsii* | Obermallebarn | 6 | Short | Absent | Present | 0.514 | 1.406 | 1.482 |  |
| ASP_50 | F | *M. robertsii* | Obermallebarn | 74 | Long | Absent | Absent | 2.468 | 2.349 | 1.287 |  |
| ASP_51 | F | *M. robertsii* | Obermallebarn | 7 | Short | Present | Present | 0.000 | 0.000 | 0.000 |  |
| ASP_52 | F | *M. robertsii* | Obermallebarn | 23 | Medium | Absent | Absent | 1.361 | 1.250 | 1.479 |  |
| ASP_53 | F | *M. robertsii* | Obermallebarn | 45 | Medium | Absent | Absent | 1.657 | 1.583 | 1.582 |  |
| ASP_54 | F | *M. robertsii* | Obermallebarn | 7 | Short | Present | Present | 0.000 | 0.000 | 0.000 |  |
| ASP_55 | M | *M. robertsii* | Obermallebarn | 70 | Long | Absent | Absent | 1.476 | 1.358 | 1.007 |  |

**Supplementary Table S1** Alpha diversity (Shannon index) values of fungal ITS and bacterial 16S data.

| **Intestinal samples** | | **Sex** | **Treatment** | **Location** | **Days until  death** | **Survival  group** | **Internal mycosis** | **External mycosis** | **Shannon index  ITS with  *Metarhizium* spp.** | **Shannon index  ITS without  *Metarhizium* spp.** | **Shannon index  16S** |
| --- | --- | --- | --- | --- | --- | --- | --- | --- | --- | --- | --- |
| ASP_56 | F | *M. robertsii* | Rückersdorf | 9 | Short | Present | Present | 0.054 | 0.000 | 1.473 |  |
| ASP_57 | M | *M. robertsii* | Rückersdorf | 9 | Short | Present | Present | 0.051 | 0.000 | 1.641 |  |
| ASP_58 | F | *M. robertsii* | Rückersdorf | 7 | Short | Present | Present | 0.000 | 0.000 | 0.635 |  |
| ASP_59 | F | *M. robertsii* | Rückersdorf | 35 | Medium | Absent | Absent | 1.179 | 0.972 | 1.960 |  |
| ASP_60 | F | *M. robertsii* | Rückersdorf | 8 | Short | Present | Present | 0.000 | 0.000 | 0.152 |  |
| ASP_61 | F | *M. robertsii* | Rückersdorf | 70 | Long | Absent | Absent | 1.961 | 1.961 | 1.529 |  |
| ASP_62 | F | *M. robertsii* | Rückersdorf | 77 | Long | Absent | Absent | 1.415 | 1.414 | 1.211 |  |
| ASP_63 | M | *M. robertsii* | Rückersdorf | 5 | Short | Absent | Present | 0.000 | 0.000 | NA |  |
| ASP_64 | F | *M. robertsii* | Ruppersthal | 39 | Medium | Absent | Absent | 1.695 | 1.499 | 0.359 |  |
| ASP_65 | F | *M. robertsii* | Ruppersthal | 63 | Long | Absent | Absent | 1.618 | 1.571 | 1.713 |  |
| ASP_66 | M | *M. robertsii* | Ruppersthal | 7 | Short | Present | Present | 0.086 | 0.340 | 0.861 |  |
| ASP_67 | F | *M. robertsii* | Ruppersthal | 30 | Medium | Present | Present | 0.000 | 0.000 | 0.866 |  |
| ASP_68 | M | *M. robertsii* | Ruppersthal | 9 | Short | Present | Present | 0.085 | 0.000 | 0.618 |  |
| ASP_69 | F | *M. robertsii* | Ruppersthal | 3 | Short | Present | Present | 0.000 | 0.000 | 1.023 |  |
| ASP_70 | F | *M. robertsii* | Ruppersthal | 98 | Long | Absent | Absent | 0.531 | 0.485 | 0.621 |  |


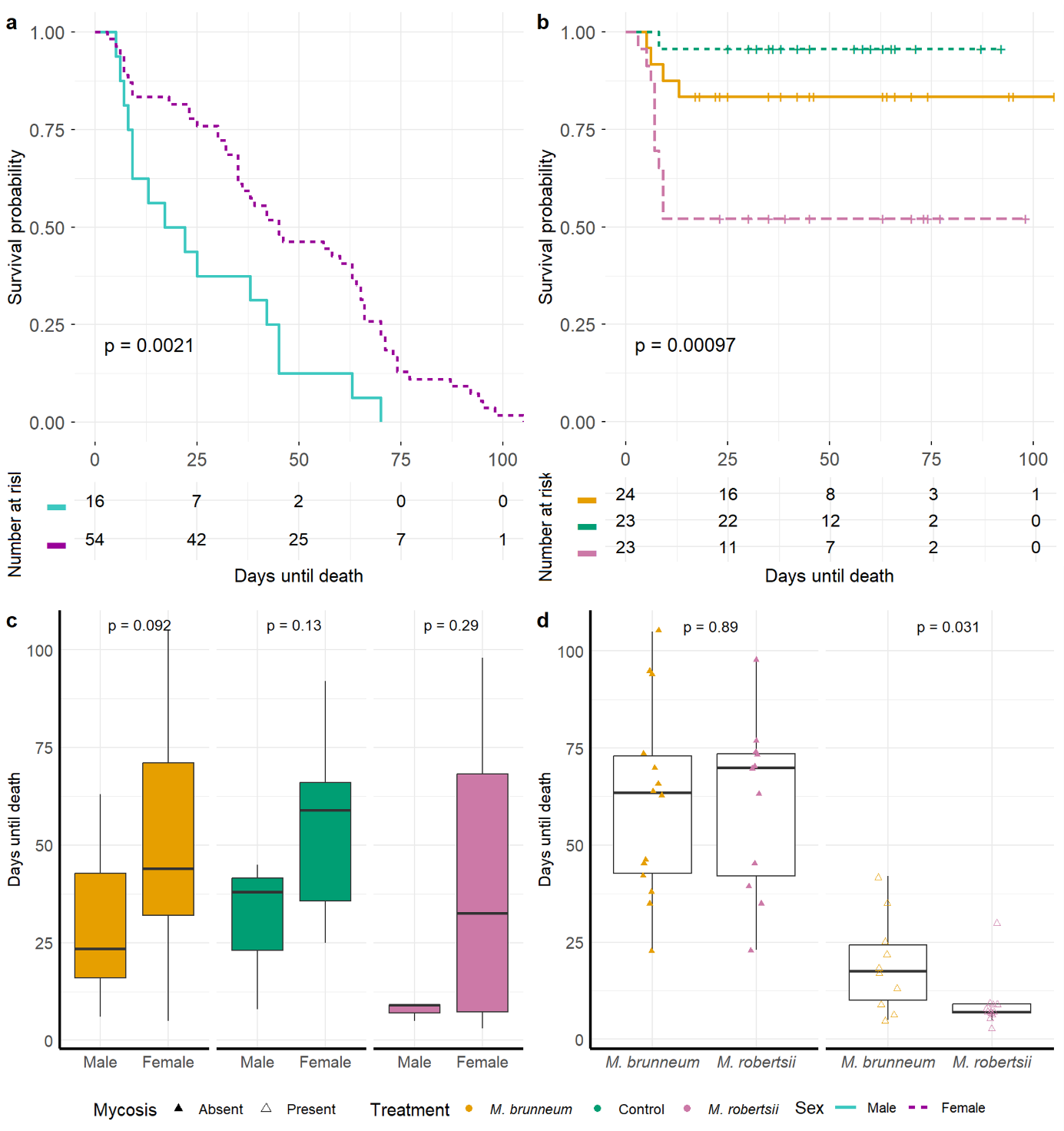


**Figure S1** Bioassay results of the individuals used in the microbiome analysis. Survival probability [%] based on KM tests is shown in line graphs comparing sex (turquoise: male; purple: female) (a) and treatment groups (yellow: *M. brunneum*; pink: *M. robertsii*; green: control group) (b). Mean lifespan (in days) of each SBW based on the treatment group and sex (c), and the presence or absence of a mycosis (filled triangle: non-mycotic; empty triangle: mycotic) caused either by *M. brunneum* or *M. robertsii* were determined (d). Significant differences in mean survival were determined using KW tests. NS = not significant, * p ≤ 0.1, ** p ≤ 0.05, *** p ≤ 0.01


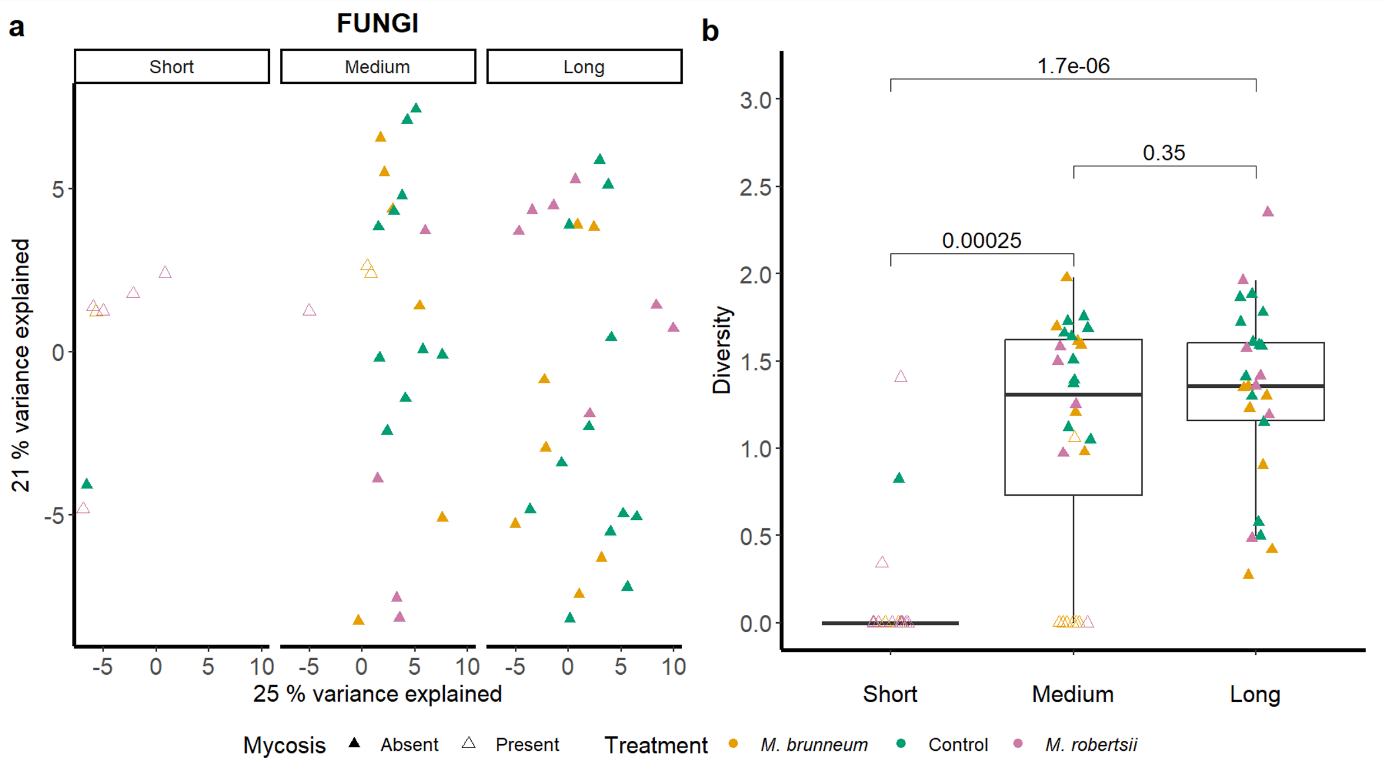


**Figure S2** Fungal beta (a) and alpha diversity (b) of the intestinal microbiome of SBWs excluding the genus Metarhizium. Diversity was analysed based on survival spans of SBWs (short: ≤ ten days; medium: ≤ 50 days; long: > 50 days), treatments (yellow: M. brunneum; pink: M. robertsii; green: control group), and the presence or absence of mycosis (filled triangle: non-mycotic; empty triangle: mycotic). RPCA shows clustering of samples with similar compositions. Significance was calculated via PERMANOVA (a). Alpha diversity was measured with the Shannon’s diversity index. Significance in alpha diversity was determined by Wilcoxon tests (b). NS = not significant, * p ≤ 0.1, ** p ≤ 0.05, *** p ≤ 0.01

**Supplementary Table S2** Wilcoxon tests of the factors "Survival", "Treatment" and "Mycosis" using the bacterial (16S) and fungal (ITS) datasets. The interaction "Mycosis and Survival" was tested on SBWs that survived ten to 50 days (group involved mycotic and non-mycotic individuals).

| **Dataset** | **Factor** | **Variable 1** | **Variable 2** | ***P*-value** | **Significance^a^** |
| --- | --- | --- | --- | --- | --- |
| ITS with *Metarhizium* spp. | Survival | Short | Medium | 0.000 | *** |
|  |  | Short | Long | 0.000 | *** |
|  |  | Medium | Long | 0.363 | NS |
| ITS without *Metarhizium* spp. | Survival | Short | Medium | 0.000 | *** |
|  |  | Short | Long | 0.000 | *** |
|  |  | Medium | Long | 0.345 | NS |
| 16S | Survival | Short | Medium | 0.012 | ** |
|  |  | Short | Long | 0.001 | *** |
|  |  | Medium | Long | 0.114 | NS |
| ITS with *Metarhizium* spp. | Treatment | *M. brunneum* | Control | 0.001 | *** |
|  |  | *M. brunneum* | *M. robertsii* | 0.929 | NS |
|  |  | *M. robertsii* | Control | 0.002 | *** |
| ITS without *Metarhizium* spp. | Treatment | *M. brunneum* | Control | 0.001 | *** |
|  |  | *M. brunneum* | *M. robertsii* | 0.964 | NS |
|  |  | *M. robertsii* | Control | 0.001 | *** |
| 16S | Treatment | *M. brunneum* | Control | 0.761 | NS |
|  |  | *M. brunneum* | *M. robertsii* | 0.104 | NS |
|  |  | *M. robertsii* | Control | 0.335 | NS |
| ITS with *Metarhizium* spp.^b^ | Mycosis | Present | Absent | 0.000 | *** |
| ITS without *Metarhizium* spp.^b^ | Mycosis | Present | Absent | 0.000 | *** |
| 16S^b^ | Mycosis | Present | Absent | 0.002 | *** |
| ITS with *Metarhizium* spp.^b^ | Mycosis:Survival | Present_Medium | Absent_Medium | 0.000 | *** |
| ITS without *Metarhizium* spp.^b^ | Mycosis:Survival | Present_Medium | Absent_Medium | 0.001 | *** |
| 16S^b^ | Mycosis:Survival | Present_Medium | Absent_Medium | 0.237 | NS |

^a^) NS = not significant, * p ≤ 0.1, ** p ≤ 0.05, *** p ≤ 0.01.

^b^) Tests in which the control group was not included.

**Supplementary Table S3** PERMANOVA of fungal (ITS) and bacterial (16S) data.

| **Dataset** | **Factor** | **Df** | **SumOfSqs** | **R^2^** | **F** | ***P*-value** | **Significance^a^** |
| --- | --- | --- | --- | --- | --- | --- | --- |
| ITS | **Treatment** | 2 | 1.406 | 0.077 | 3.449 | 0.002 | *** |
|  | **Survival** | 2 | 2.708 | 0.147 | 6.643 | 0.001 | *** |
|  | **Mycosis** | 1 | 1.423 | 0.077 | 6.983 | 0.001 | *** |
|  | **Residual** | 63 | 12.840 | 0.699 | NA | NA |  |
| 16S | **Treatment** | 2 | 0.735 | 0.053 | 2.096 | 0.018 | ** |
|  | **Survival** | 2 | 1.928 | 0.139 | 5.500 | 0.001 | *** |
|  | **Mycosis** | 1 | 0.359 | 0.026 | 2.048 | 0.044 | ** |
|  | **Residual** | 62 | 10.867 | 0.782 | NA | NA |  |

^a^) NS = not significant, * p ≤ 0.1, ** p ≤ 0.05, *** p ≤ 0.01.

**Supplementary Table S4** Log2 fold-change (log2FC) coefficients from the Wald test and p-values of the indicator taxa for the presence or absence of a mycosis in treated SBWs calculated via DESeq2 analysis.

| **Taxon** | **External mycosis^a^** | **baseMean** | **log2FC** | **lfcSE** | **stat** | ***P*-value** | ***P*-value adj.** |
| --- | --- | --- | --- | --- | --- | --- | --- |
| *Metarhizium* | Present | 571.025 | -8.662 | 1.855 | -4.669 | 0.000 | 0.000 |
| *Cladosporium* | Absent | 443.984 | 6.960 | 1.482 | 4.697 | 0.000 | 0.000 |
| *Mortierella* | Absent | 48.455 | 7.260 | 1.706 | 4.255 | 0.000 | 0.000 |
| *Botryotrichum* | Absent | 55.770 | 26.311 | 1.963 | 13.404 | 0.000 | 0.000 |
| *Cephalotrichum* | Absent | 512.556 | 29.566 | 1.704 | 17.356 | 0.000 | 0.000 |
| *Pantoea* | Present | 46.370 | -26.545 | 2.617 | -10.145 | 0.000 | 0.000 |
| *Salmonella* | Absent | 856.515 | 6.625 | 1.649 | 4.017 | 0.000 | 0.000 |
| *Sphingobacterium* | Absent | 16.471 | 23.192 | 2.956 | 7.846 | 0.000 | 0.000 |
| *Serratia* | Absent | 12.973 | 24.224 | 2.956 | 8.195 | 0.000 | 0.000 |
| *Stenotrophomonas* | Absent | 26.404 | 25.192 | 2.955 | 8.524 | 0.000 | 0.000 |
| *Staphylococcus* | Absent | 46.492 | 26.011 | 2.955 | 8.802 | 0.000 | 0.000 |
| **Taxon** | **Internal mycosis^a^** | **baseMean** | **log2FC** | **lfcSE** | **stat** | ***P*-value** | ***P*-value adj.** |
| *Metarhizium* | Present | 501.192 | -5.748 | 2.485 | -2.313 | 0.021 | 0.033 |
| *Mortierella* | Absent | 48.455 | 25.437 | 2.274 | 11.184 | 0.000 | 0.000 |
| *Botryotrichum* | Absent | 55.770 | 25.624 | 2.533 | 10.118 | 0.000 | 0.000 |
| *Cladosporium* | Absent | 443.984 | 26.371 | 1.764 | 14.947 | 0.000 | 0.000 |
| *Cephalotrichum* | Absent | 512.556 | 28.735 | 2.280 | 12.603 | 0.000 | 0.000 |
| *Pantoea* | Absent | 7.621 | 23.030 | 3.250 | 7.087 | 0.000 | 0.000 |
| *Serratia* | Absent | 12.973 | 23.776 | 3.249 | 7.318 | 0.000 | 0.000 |
| *Sphingobacterium* | Absent | 16.471 | 24.020 | 3.248 | 7.394 | 0.000 | 0.000 |
| *Stenotrophomonas* | Absent | 26.404 | 24.770 | 3.248 | 7.626 | 0.000 | 0.000 |
| *Staphylococcus* | Absent | 46.492 | 25.560 | 3.248 | 7.870 | 0.000 | 0.000 |
| *Salmonella* | Absent | 1004.758 | 29.865 | 1.689 | 17.680 | 0.000 | 0.000 |

^a^) The absence of a mycosis i.e. non-mycotic SBWs were set as reference.

**Supplementary Table S5** Coefficients and Benjamini-Hochberg-corrected p-values of indicator taxa for the presence or absence of a mycosis calculated via MaAsLin2 analysis.

| **Treatment** | **Taxon** | **coeff** | **stderr** | **No. of  treated SBWs** | **Extenal mycosis^a^** | **No. of SBWs in which  the taxon is non-zero** | ***P*-value** | ***P*-value  adj.** |
| --- | --- | --- | --- | --- | --- | --- | --- | --- |
| *M. robertsii* | *Metarhizium* | 4.968 | 0.861 | 23 | Present | 20 | 0.000 | 0.000 |
|  | *Penicillium expansum* | -7.929 | 2.635 | 23 | Absent | 11 | 0.003 | 0.017 |
| *M. brunneum* | *Enterobacteriaceae* | -2.325 | 0.048 | 23 | Absent | 14 | 0.000 | 0.000 |
|  | *Salmonella* | 3.608 | 0.039 | 23 | Present | 11 | 0.000 | 0.000 |
|  | *Erwiniaceae* | 3.907 | 0.246 | 23 | Present | 6 | 0.000 | 0.000 |
|  | *Serratia* | -10.831 | 3.338 | 23 | Absent | 5 | 0.001 | 0.003 |
|  | *Stenotrophomonas* | -5.300 | 1.997 | 23 | Absent | 6 | 0.008 | 0.003 |
|  | *Cephalotrichum stemonitis* | -10.929 | 2.310 | 23 | Absent | 13 | 0.000 | 0.000 |
| **Treatment** | **Taxon** | **coeff** | **stderr** | **No. of  treated SBWs** | **Internal mycosis^a^** | **No. of SBWs in which  the taxon is non-zero** | ***P*-value** | ***P*-value  adj.** |
| *M. brunneum* | *Enterobacteriaceae* | -2.326 | 0.048 | 23 | Absent | 14 | 0.000 | 0.000 |
|  | *Erwiniaceae* | 3.915 | 0.247 | 23 | Present | 6 | 0.000 | 0.000 |

^a^) The absence of a mycosis i.e. non-mycotic SBWs were set as reference.


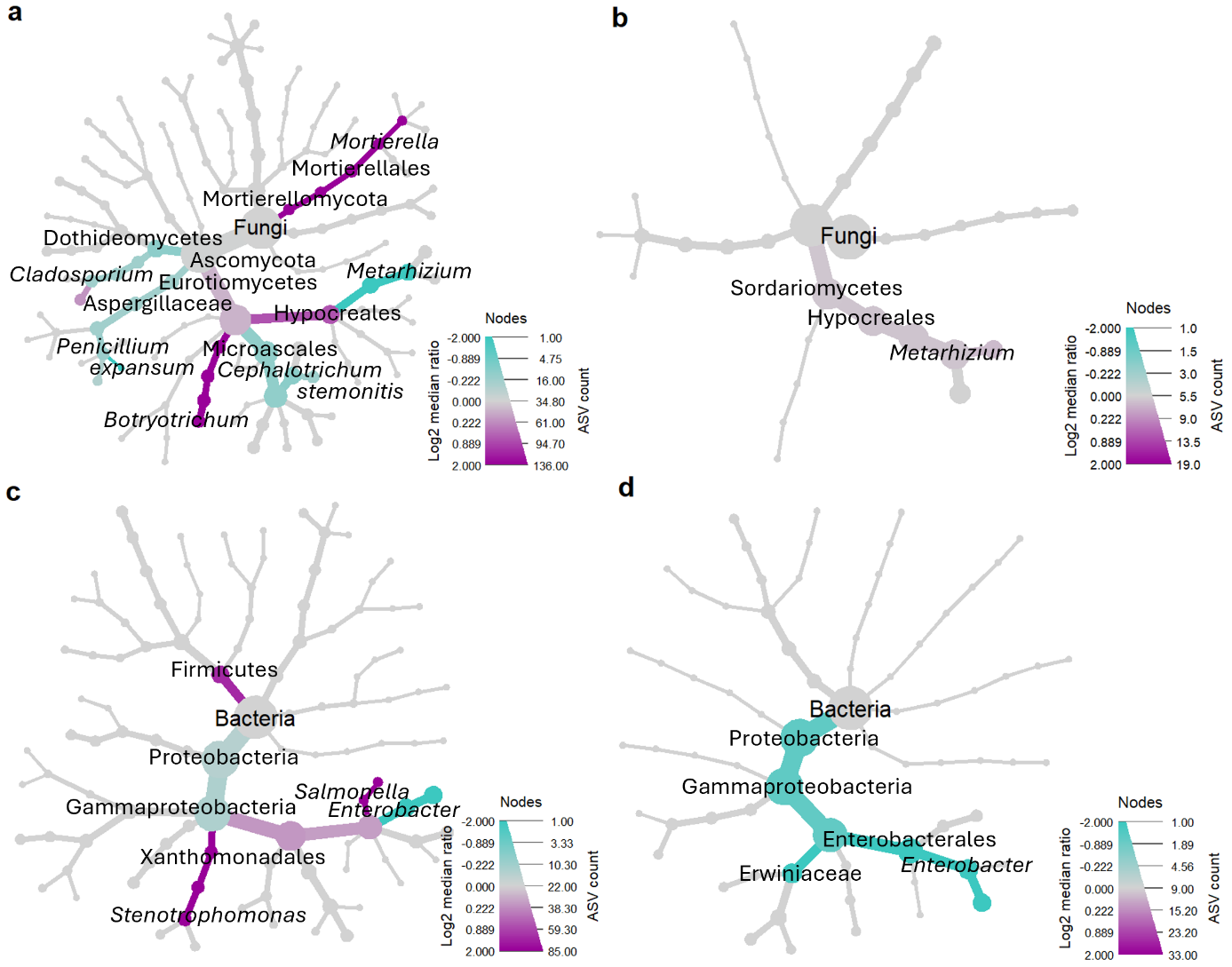


**Figure S3** Differences in microbial composition in male and female SBWs. Fungal (a, b) and bacterial (c, d) composition in the intestine of weevils treated with *M. brunneum* and *M. robertsii* (excluding the control group). Different taxonomic ranks are shown in nodes, starting from the highest rank (largest nodes; great numbers of ASVs assigned to the taxon) to the genus or species level at the end of the branches. Coloured nodes, based on the log2 median ratio proportion [-2;2], represent taxa which are more enriched in male (turquoise) or female (purple) individuals. Significant taxa (Wilcoxon p < 0.05) are highlighted
